# Supplementary material for: 24-hour movement behaviours and mental health in non-clinical populations: A systematic review
Source: PLoS One. 2025 Jun 9;20(6):e0325445. doi: 10.1371/journal.pone.0325445 (PMC12148175; doi:10.1371/journal.pone.0325445)
Supplement: S1 File — S1 Methods. S1 Table. PubMed search strategy. S1 Results. Further details on sub-groups and analysis types. S2 Table. summary of findings in children. S3 Table. summary of findings in adolescents. Supplementary table S4: summary of findings in adults. (DOCX) [file pone.0325445.s001.docx]

24-hour movement behaviours & mental health in non-clinical populations; a systematic review.

Supplementary methods:

Movement behaviours are based on the definitions used by the individual studies. Typically:

Typically:

• Sleep: hours of sleep or hours in bed

• Sedentary time: <1.5 metabolic equivalents (METs) during waking hours

• Screen time: time spent in screen-based activities

• Muscle strengthening: number of days per week

• LPA: 1.5-2.9 METs

• MVPA: >3.0 METs

Supplementary tables S1-S4

Table S1: PubMed Search strategy

| Search number | Query | Search Details | Results (15.2.22) |
| --- | --- | --- | --- |
| 8 | #6 AND #7 | ("24-hour movement"[Title/Abstract] OR "24-hr movement"[Title/Abstract] OR "movement behavio*"[Title/Abstract] OR "movement guideline*"[Title/Abstract] OR (("Sleep"[MeSH Terms] OR "Circadian Rhythm"[MeSH Terms] OR "sleep*"[Title/Abstract]) AND ("Sedentary Behavior"[MeSH Terms] OR "Screen Time"[MeSH Terms] OR "sedentary"[Title/Abstract] OR "Screen Time"[Title/Abstract] OR "television"[Title/Abstract] OR "computer"[Title/Abstract]) AND ("Exercise"[MeSH Terms] OR "Sports"[MeSH Terms] OR "physical exercis*"[Title/Abstract] OR "sport*"[Title/Abstract] OR "physical activ*"[Title/Abstract] OR "activity"[Title] OR "Exercise"[Title]))) AND ("Mental Health"[MeSH Terms] OR "Depression"[MeSH Terms] OR "Depressive Disorder"[MeSH Terms] OR "Psychosocial Functioning"[MeSH Terms] OR "Emotions"[MeSH Terms] OR "mental*"[Title/Abstract] OR "depress*"[Title/Abstract] OR "anxiety"[Title/Abstract] OR "wellbeing"[Title/Abstract] OR "well-being"[Title/Abstract] OR "psychosocial"[Title/Abstract]) | 683 |
| 7 | "Mental Health"[Mesh] OR "Depression"[Mesh] OR "Depressive Disorder"[Mesh] OR "Psychosocial Functioning"[Mesh] OR "Emotions"[Mesh] OR mental*[Title/Abstract] OR depress*[Title/Abstract] OR anxiety[Title/Abstract] OR wellbeing[Title/Abstract] OR well-being[Title/Abstract] OR psychosocial[Title/Abstract] | "Mental Health"[MeSH Terms] OR "Depression"[MeSH Terms] OR "Depressive Disorder"[MeSH Terms] OR "Psychosocial Functioning"[MeSH Terms] OR "Emotions"[MeSH Terms] OR "mental*"[Title/Abstract] OR "depress*"[Title/Abstract] OR "anxiety"[Title/Abstract] OR "wellbeing"[Title/Abstract] OR "well-being"[Title/Abstract] OR "psychosocial"[Title/Abstract] | 1,310,709 |
| 6 | #1 OR #5 | "24-hour movement"[Title/Abstract] OR "24-hr movement"[Title/Abstract] OR "movement behavio*"[Title/Abstract] OR "movement guideline*"[Title/Abstract] OR (("Sleep"[MeSH Terms] OR "Circadian Rhythm"[MeSH Terms] OR "sleep*"[Title/Abstract]) AND ("Sedentary Behavior"[MeSH Terms] OR "Screen Time"[MeSH Terms] OR "sedentary"[Title/Abstract] OR "Screen Time"[Title/Abstract] OR "television"[Title/Abstract] OR "computer"[Title/Abstract]) AND ("Exercise"[MeSH Terms] OR "Sports"[MeSH Terms] OR "physical exercis*"[Title/Abstract] OR "sport*"[Title/Abstract] OR "physical activ*"[Title/Abstract] OR "activity"[Title] OR "Exercise"[Title])) | 3,955 |
| 5 | #2 AND #3 AND #4 | ("Sleep"[MeSH Terms] OR "Circadian Rhythm"[MeSH Terms] OR "sleep*"[Title/Abstract]) AND ("Sedentary Behavior"[MeSH Terms] OR "Screen Time"[MeSH Terms] OR "sedentary"[Title/Abstract] OR "Screen Time"[Title/Abstract] OR "television"[Title/Abstract] OR "computer"[Title/Abstract]) AND ("Exercise"[MeSH Terms] OR "Sports"[MeSH Terms] OR "physical exercis*"[Title/Abstract] OR "sport*"[Title/Abstract] OR "physical activ*"[Title/Abstract] OR "activity"[Title] OR "Exercise"[Title]) | 2,519 |
| 4 | "Exercise"[Mesh] OR "Sports"[Mesh] OR "physical exercis*"[Title/Abstract] OR sport*[Title/Abstract] OR "physical activ*"[Title/Abstract] OR activity[Title] OR exercise[Title] | "Exercise"[MeSH Terms] OR "Sports"[MeSH Terms] OR "physical exercis*"[Title/Abstract] OR "sport*"[Title/Abstract] OR "physical activ*"[Title/Abstract] OR "activity"[Title] OR "Exercise"[Title] | 1,055,685 |
| 3 | "Sedentary Behavior"[Mesh] OR "Screen Time"[Mesh] OR "sedentary"[Title/Abstract] OR "screen time"[Title/Abstract] OR television[Title/Abstract] OR computer[Title/Abstract] | "Sedentary Behavior"[MeSH Terms] OR "Screen Time"[MeSH Terms] OR "sedentary"[Title/Abstract] OR "Screen Time"[Title/Abstract] OR "television"[Title/Abstract] OR "computer"[Title/Abstract] | 291,724 |
| 2 | "Sleep"[Mesh] OR "Circadian Rhythm"[Mesh] OR sleep*[Title/Abstract] | "Sleep"[MeSH Terms] OR "Circadian Rhythm"[MeSH Terms] OR "sleep*"[Title/Abstract] | 285,540 |
| 1 | "24-hour movement"[Title/Abstract] OR "24-hr movement"[Title/Abstract] OR "movement behavio*"[Title/Abstract] OR "movement guideline*"[Title/Abstract] | "24-hour movement"[Title/Abstract] OR "24-hr movement"[Title/Abstract] OR "movement behavio*"[Title/Abstract] OR "movement guideline*"[Title/Abstract] | 1,721 |

Supplementary results:

Within the adult sub-group, there was heterogeneity in the sample populations: general population n=10 studies, university students/young adults n=11, over 45-year-olds n=2, over 60s n=6, office workers n=3, inactive people n=2, cancer survivors n=2, pre-school caregivers n=1, nurses n=1, military personnel n=1, preconception/recently pregnant n=1, visually impaired n=1, priests n=1.

Many of the studies were conducted in Canada (n=28) and China (n=28), followed by the USA (n=17) and Australia (n=8), with the UK (n=5), Spain (n=4), Japan (n=3), Brazil (n=2), Germany (n=1), Switzerland (n=1), New Zealand (n=1), the Netherlands (n=1), Slovenia (n=1), Singapore (n=1), Bangladesh (n=1), Inner Mongolia (n=1) and Sweden (n=1) also represented. South America and Africa are currently highly underrepresented in this field of research.

Analysing the associations between meeting the guidelines, or individual recommendations (whilst taking the other recommendations into account) and/or combinations of different recommendations and mental health was the most common method (n=57 studies) and was typically done using linear regression models. In addition, compositional data analysis (CoDA), which accounts for the minutes per day spent in each activity, was also a common method (n=25 studies). Isotemporal substitution analyses (ISM) model the effect of replacing certain movement behaviours with others on mental health outcomes and was employed by 32 studies. Finally, some studies (n=7) also used techniques to group participants into categories, e.g. high MVPA/low ST, and analyse the mental health differences between these groups (latent profile analyses n=4, cluster analyses n=1, principal components analyses n=1, composite activity-sleep score n=1).

Results summary tables

Table S2: findings summary children (n=27)

| Outcome | Analyses | Positive associations with mental health | Negative associations with mental health | No significant finding |
| --- | --- | --- | --- | --- |
| Emotional problems/internalising/externalising  n=18  (Bang et al., 2020; Bao et al., 2024; Brown, Kwan, King-Dowling, et al., 2021; Carson et al., 2019; Fairclough et al., 2021, 2023; Fung et al., 2023; Hinkley et al., 2020; Hou et al., 2024; Kuzik et al., 2020; Li et al., 2024; López-Gil et al., 2022; Mcneill et al., 2020; Rorem et al., 2024; Sampasa-Kanyinga, Colman, Goldfield, et al., 2021; Y. Sun et al., 2024; Yin et al., 2024; N. Zhu et al., 2023) | Meet guidelines yes/no  n=12  (Bang et al., 2020; Bao et al., 2024; Carson et al., 2019; Fung et al., 2023; Hinkley et al., 2020; Hou et al., 2024; López-Gil et al., 2022; Mcneill et al., 2020; Sampasa-Kanyinga, Colman, Goldfield, et al., 2021; Y. Sun et al., 2024; Yin et al., 2024; N. Zhu et al., 2023) | Meeting ST -> lower total, internalising n=1 (Carson et al., 2019)& externalising problems n=2 (Carson et al., 2019; Yin et al., 2024)  Meeting MVPA + ST -> lower total, internalising & externalising problems n=1 (Carson et al., 2019) Meeting ST + sleep -> lower total, internalising & externalising problems n=2 (Carson et al., 2019; Yin et al., 2024)  Meeting 0 guidelines -> higher total n=2 (Carson et al., 2019; Sampasa-Kanyinga, Colman, Goldfield, et al., 2021), externalising n=3 (Carson et al., 2019; Hou et al., 2024; Sampasa-Kanyinga, Colman, Goldfield, et al., 2021), internalising problems n=2 (Hou et al., 2024; Sampasa-Kanyinga, Colman, Goldfield, et al., 2021)  Meeting 3 guidelines -> lower emotional problems n=2 (López-Gil et al., 2022; N. Zhu et al., 2023)  Dose-response gradient n=3 (Bao et al., 2024; Fung et al., 2023; Sampasa-Kanyinga, Colman, Goldfield, et al., 2021) | Meeting 0 guidelines -> lower externalising score than meeting only MVPA n=2 (Fung et al., 2023; Sampasa-Kanyinga, Colman, Goldfield, et al., 2021) | n=4 (Bang et al., 2020; Hinkley et al., 2020; Mcneill et al., 2020; Y. Sun et al., 2024) |
|  | CoDA  n=5  (Brown, Kwan, King-Dowling, et al., 2021; Fairclough et al., 2021, 2023; Kuzik et al., 2020; Rorem et al., 2024) | More sedentary -> more externalising problems n=1 (Brown, Kwan, King-Dowling, et al., 2021)  More sedentary -> more internalising problems n=1 (Fairclough et al., 2021)  Less ST -> fewer total n=1, internalising n=1 (Rorem et al., 2024), externalising problems n=2 (Fairclough et al., 2023; Rorem et al., 2024)  More sleep -> fewer externalising problems n=1 (Fairclough et al., 2023) | More MVPA -> higher total and externalising problems n=1 (Rorem et al., 2024) | n=1  (Kuzik et al., 2020) |
|  | ISM  n=4  (Brown, Kwan, King-Dowling, et al., 2021; Fairclough et al., 2021; Li et al., 2024; Rorem et al., 2024) | Replacing sleep or MVPA with sedentary -> higher internalising problems n=1 (Fairclough et al., 2021)  Increasing ST -> higher total, internalising, externalising problems n=1 (Rorem et al., 2024) | Replacing sleep with non-screen sedentary -> lower externalising problems n=1 (Li et al., 2024) | n=1  (Brown, Kwan, King-Dowling, et al., 2021) |
| Depression  n=7  (Fairclough et al., 2021; Hansen et al., 2022; Hou et al., 2023, 2024; K. Liang et al., 2023; Taylor et al., 2021; X. Zhu et al., 2019) | Meet guidelines yes/no  n=6  (Hansen et al., 2022; Hou et al., 2023, 2024; K. Liang et al., 2023; Taylor et al., 2021; X. Zhu et al., 2019) | Meeting ST -> lower depression n=2 (Hansen et al., 2022; K. Liang et al., 2023)  Meeting MVPA -> lower depression n=3 (Hansen et al., 2022; K. Liang et al., 2023; Taylor et al., 2021) Meeting sleep -> lower depression n=2 (Hansen et al., 2022; K. Liang et al., 2023) Meeting sedentary -> lower depression n=1 (Taylor et al., 2021)  Meeting ST + sleep -> lower depression n=1 (Hou et al., 2023)  Meeting 0 guidelines -> higher depression n=2 (Hou et al., 2024; K. Liang et al., 2023) Meeting 3 guidelines -> lower depression n=3 (Hansen et al., 2022; Taylor et al., 2021; X. Zhu et al., 2019) |  |  |
|  | CoDA  n=1  (Fairclough et al., 2021) |  |  | n=1  (Fairclough et al., 2021) |
| Anxiety  n=4  (Hou et al., 2023; K. Liang et al., 2023; Taylor et al., 2021; X. Zhu et al., 2019) | Meet guidelines yes/no  n=4  (Hou et al., 2023; K. Liang et al., 2023; Taylor et al., 2021; X. Zhu et al., 2019) | Meeting MVPA -> lower anxiety n=1 (Taylor et al., 2021)  Meeting sleep -> lower anxiety n=2 (K. Liang et al., 2023; Taylor et al., 2021)  Meeting sedentary -> lower anxiety n=1 (Taylor et al., 2021)  Meeting ST -> lower anxiety n=1 (K. Liang et al., 2023)  Meeting ST + sleep -> lower anxiety n=1 (Hou et al., 2023)  Meeting 3 guidelines -> lower anxiety n=4 (Hou et al., 2023; K. Liang et al., 2023; Taylor et al., 2021; X. Zhu et al., 2019) |  |  |
| Resilience  n=3  (Hou et al., 2023; K. Liang et al., 2023; Taylor et al., 2021) | Meet guidelines yes/no  n=3  (Hou et al., 2023; K. Liang et al., 2023; Taylor et al., 2021) | Meeting sleep -> higher resilience n=1 (Taylor et al., 2021)  Meeting MVPA + ST -> higher resilience n=1 (Hou et al., 2023)  Dose-response gradient n=1 (K. Liang et al., 2023) |  |  |
| Self-esteem  n=2  (Fairclough et al., 2021; Hinkley et al., 2020) | Meet guidelines yes/no  n=1  (Hinkley et al., 2020) |  |  | n=1  (Hinkley et al., 2020) |
|  | CoDA  n=1  (Fairclough et al., 2021) |  |  | n=1  (Fairclough et al., 2021) |
| Quality of life  n=3  (Hinkley et al., 2020; Peralta et al., 2022; Tan et al., 2023) | Meet guidelines yes/no  n=2  (Hinkley et al., 2020; Peralta et al., 2022) | Dose-response gradient n=1 (Peralta et al., 2022) |  | n=1  (Hinkley et al., 2020) |
|  | CoDA & ISM  n=1  (Tan et al., 2023) |  |  | n=1  (Tan et al., 2023) |
| Mental health/general distress/diagnosis  n=3  (Kasai et al., 2024; K. Liang et al., 2023; J. Sun et al., 2023) | Meet guidelines yes/no  n=3  (Kasai et al., 2024; K. Liang et al., 2023; J. Sun et al., 2023) | Meeting MVPA -> better mental health n=1 (Kasai et al., 2024)  Meeting ST -> better mental health n=1 (Kasai et al., 2024)  Does-response gradient n=2 (K. Liang et al., 2023; J. Sun et al., 2023) |  |  |

Table S3: findings summary adolescents (n=41)

| Outcome | Analyses | Positive associations with mental health | Negative associations with mental health | No significant finding |
| --- | --- | --- | --- | --- |
| Emotional problems/ internalising/ externalising  n=11  (Bang et al., 2020; Chong et al., 2021; Dumuid et al., 2021; Duncan et al., 2022; Fairclough et al., 2021; Fung et al., 2023; Hou et al., 2024; Huang et al., 2024; Janssen et al., 2017; López-Gil et al., 2022; Y. Sun et al., 2024) | Meet guidelines yes/no  n=7  (Bang et al., 2020; Fung et al., 2023; Hou et al., 2024; Huang et al., 2024; Janssen et al., 2017; López-Gil et al., 2022; Y. Sun et al., 2024) | Meeting sleep -> lower emotional problems, n=1 (Bang et al., 2020)  Meeting ST -> lower emotional problems, n=1 (Bang et al., 2020)  Meeting MVPA-> lower emotional problems, n=1 (Bang et al., 2020)  Meeting 0 guidelines -> higher emotional problems, n=2 (Hou et al., 2024; Janssen et al., 2017)  Meeting ≥2 guidelines ->lower emotional problems, n=1 (Bang et al., 2020)  Meeting 3 guidelines -> lower emotional problems, n=1 (López-Gil et al., 2022)  Dose-response gradient n=3 (Fung et al., 2023; Huang et al., 2024; Janssen et al., 2017) | Meeting 0 guidelines -> lower externalising score than meeting only MVPA n=1 (Fung et al., 2023) | n=1 (Y. Sun et al., 2024) |
|  | CoDA  n=3  (Chong et al., 2021; Dumuid et al., 2021; Fairclough et al., 2021) | Higher sedentary ->higher total n=1 (Chong et al., 2021), higher internalising, n=2 (Fairclough et al., 2021)  Higher sleep ->lower total, n=2 (Chong et al., 2021; Dumuid et al., 2021), lower internalising, n=1 (Chong et al., 2021)  Higher LPA-> lower total (Chong et al., 2021), lower internalising, n=1 (Chong et al., 2021)  Higher ST-> higher total, higher internalising, higher externalising, n=1 (Chong et al., 2021)  Higher MVPA-> lower total problems, n=1 (Dumuid et al., 2021) |  |  |
|  | ISM  n=2  (Duncan et al., 2022; Fairclough et al., 2021) | Replacing sleep or MVPA with sedentary -> higher internalising score n=1 (Fairclough et al., 2021)  Replacing ST with MVPA or sleep -> lower emotional problems n=1 (Duncan et al., 2022) |  |  |
| Depression  n=23  (Brown, Kwan, Arbour-Nicitopoulos, et al., 2021; Burns et al., 2020; Cao et al., 2020; de Faria et al., 2022; Dumuid et al., 2021; Duncan et al., 2022, 2024; Fairclough et al., 2021; Gilchrist et al., 2021; Hansen et al., 2022; Hou et al., 2023; Khan et al., 2024; Liang et al., 2023; López-Gil et al., 2024; Lu et al., 2021; Luo et al., 2023; Monteagudo et al., 2023; Patte et al., 2020; Sampasa-Kanyinga, Chaput, et al., 2021; Sampasa-Kanyinga, Colman, Dumuid, et al., 2021; Yuan et al., 2023; Y. Zhang et al., 2023; X. Zhu et al., 2019) | Meet guidelines yes/no  n=13  (Burns et al., 2020; Hansen et al., 2022; Hou et al., 2023; Khan et al., 2024; Liang et al., 2023; López-Gil et al., 2024; Lu et al., 2021; Luo et al., 2023; Patte et al., 2020; Sampasa-Kanyinga, Chaput, et al., 2021; Yuan et al., 2023; Y. Zhang et al., 2023; X. Zhu et al., 2019) | Meeting sleep -> lower depression, n=4 (Hansen et al., 2022; Khan et al., 2024; Luo et al., 2023; Patte et al., 2020)  Meeting ST -> lower depression, n=2 (Hansen et al., 2022; Patte et al., 2020)  Meeting MVPA-> lower depression, n=2 (Hansen et al., 2022; Khan et al., 2024)  Meet sleep + ST -> lower depression, n=5 (Hou et al., 2023; Lu et al., 2021; Luo et al., 2023; Sampasa-Kanyinga, Chaput, et al., 2021; Yuan et al., 2023)  Meet MVPA + sleep -> lower depression n=2 (Khan et al., 2024; Luo et al., 2023)  Meet MVPA + ST -> lower depression n=1 (Khan et al., 2024)  Meeting 0 guidelines -> higher depression, n=5 (Burns et al., 2020; Khan et al., 2024; K. Liang et al., 2023; Patte et al., 2020; Sampasa-Kanyinga, Chaput, et al., 2021)  Meeting 3 guidelines-> lower depression, n=4 (Hansen et al., 2022; López-Gil et al., 2024; Luo et al., 2023; X. Zhu et al., 2019)  Dose-response gradient n=5 (Burns et al., 2020; Liang et al., 2023; Lu et al., 2021; Yuan et al., 2023; Y. Zhang et al., 2023) | Meeting MVPA only -> increase in depression n=1 (Yuan et al., 2023) |  |
|  | CoDA  n=5  (de Faria et al., 2022; Dumuid et al., 2021; Duncan et al., 2024; Fairclough et al., 2021; Sampasa-Kanyinga, Colman, Dumuid, et al., 2021) | Higher sleep -> lower depression, n=3 (Dumuid et al., 2021; Duncan et al., 2024; Sampasa-Kanyinga, Colman, Dumuid, et al., 2021)  Lower sedentary -> lower depression, n=2 (de Faria et al., 2022; Dumuid et al., 2021)  Higher MVPA -> lower depression, n=3 (Dumuid et al., 2021; Duncan et al., 2024; Sampasa-Kanyinga, Colman, Dumuid, et al., 2021)  Lower ST -> lower depression n=2 (Duncan et al., 2024; Sampasa-Kanyinga, Colman, Dumuid, et al., 2021)  Higher LPA -> lower depression n=1 (de Faria et al., 2022) |  | n=1  (Fairclough et al., 2021) |
|  | Latent profiles/ cluster  n=2  (Brown, Kwan, Arbour-Nicitopoulos, et al., 2021; Cao et al., 2020) | High MVPA/low ST -> lower depression, n=2 (Brown, Kwan, Arbour-Nicitopoulos, et al., 2021; Cao et al., 2020) |  |  |
|  | ISM  n=4  (Duncan et al., 2022; Gilchrist et al., 2021; Monteagudo et al., 2023; Sampasa-Kanyinga, Colman, Dumuid, et al., 2021) | Replacing ST with sleep-> lower depression, n=3 (Duncan et al., 2022; Gilchrist et al., 2021; Sampasa-Kanyinga, Colman, Dumuid, et al., 2021)  Replacing ST with MVPA-> lower depression, n=2 (Duncan et al., 2022; Sampasa-Kanyinga, Colman, Dumuid, et al., 2021)  Replacing MVPA with sleep->lower depression, n=2 (Gilchrist et al., 2021; Sampasa-Kanyinga, Colman, Dumuid, et al., 2021) |  | n=1 (Monteagudo et al., 2023) |
| Anxiety  n=13  (de Faria et al., 2022; Duncan et al., 2022, 2024; Gilchrist et al., 2021; Hou et al., 2023; Liang et al., 2023; Lu et al., 2021; Luo et al., 2023; Monteagudo et al., 2023; Sampasa-Kanyinga, Chaput, et al., 2021; Y. Zhang et al., 2023; Zhou et al., 2024; X. Zhu et al., 2019) | Meet guidelines yes/no  n=8  (Hou et al., 2023; Liang et al., 2023; Lu et al., 2021; Luo et al., 2023; Sampasa-Kanyinga, Chaput, et al., 2021; Y. Zhang et al., 2023; Zhou et al., 2024; X. Zhu et al., 2019) | Meeting sleep -> lower anxiety n=2 (Luo et al., 2023; Zhou et al., 2024)  Meeting sedentary -> lower anxiety n=1 (Zhou et al., 2024)  Meeting ST + sleep -> lower anxiety, n=4 (Hou et al., 2023; Lu et al., 2021; Luo et al., 2023; Sampasa-Kanyinga, Chaput, et al., 2021)  Meeting MVPA + sleep -> lower anxiety n=1 (Luo et al., 2023)  Meeting sedentary + sleep -> lower anxiety n=1 (Zhou et al., 2024)  Meeting 0 guidelines -> higher anxiety, n=3 (K. Liang et al., 2023; Sampasa-Kanyinga, Chaput, et al., 2021; Zhou et al., 2024)  Meeting 3 guidelines-> lower anxiety, n=2 (Luo et al., 2023; X. Zhu et al., 2019)  Dose-response gradient, n=3 (Liang et al., 2023; Lu et al., 2021; Y. Zhang et al., 2023) |  |  |
|  | CoDA  n=2  (de Faria et al., 2022; Duncan et al., 2024) | Higher sleep -> lower anxiety n=1 (Duncan et al., 2024)  Higher MVPA -> lower anxiety n=1 (Duncan et al., 2024)  Higher LPA -> lower anxiety n=1 (de Faria et al., 2022)  Lower ST -> lower anxiety n=1 (Duncan et al., 2024)  Lower sedentary -> lower anxiety n=1 (de Faria et al., 2022) |  |  |
|  | ISM  n=3  (Duncan et al., 2022; Gilchrist et al., 2021; Monteagudo et al., 2023) | Replacing ST with sleep -> lower anxiety, n=2 (Duncan et al., 2022; Gilchrist et al., 2021)  Replacing MVPA with sleep -> lower anxiety, n=1  (Gilchrist et al., 2021)  Replacing ST with MVPA -> lower anxiety n=1 (Duncan et al., 2022)  Replacing sedentary or LPA with MVPA -> lower anxiety n=1 (Monteagudo et al., 2023) | Replacing sedentary with LPA -> higher anxiety n=1 (Monteagudo et al., 2023) |  |
| Resiliency  n=5  (Brown, Cairney, et al., 2021; Brown & Kwan, 2021; Duncan et al., 2024; Hou et al., 2023; Liang et al., 2023) | Meet guidelines yes/no  n=2  (Hou et al., 2023; K. Liang et al., 2023) | Meeting MVPA + ST -> higher resiliency n=1 (Hou et al., 2023)  Dose-response gradient n=1 (K. Liang et al., 2023) |  |  |
|  | CoDA  n=1  (Duncan et al., 2024) | Higher sleep -> lower anxiety n=1 (Duncan et al., 2024)  Higher MVPA -> lower anxiety n=1 (Duncan et al., 2024)  Lower ST -> lower anxiety n=1 (Duncan et al., 2024) |  |  |
|  | Latent profiles  n=1  (Brown, Cairney, et al., 2021) | High MVPA/low ST -> better resiliency, n=1  Low MVPA/low ST -> better resiliency, n=1  High MVPA/high ST -> better resiliency, n=1  (Brown, Cairney, et al., 2021) |  |  |
|  | ISM  n=1  (Brown & Kwan, 2021) | Replacing ST with sleep or MVPA-> better resiliency, n=1 (Brown & Kwan, 2021) |  |  |
| Self-esteem  n=4  (Brown, Cairney, et al., 2021; Brown & Kwan, 2021; Fairclough et al., 2021; Sampasa-Kanyinga et al., 2022a) | Meet guidelines yes/no  n=1  (Sampasa-Kanyinga et al., 2022a) | Meeting 3 guidelines -> better self-esteem n=1 (Sampasa-Kanyinga et al., 2022a)  Dose-response gradient n=1 (Sampasa-Kanyinga et al., 2022a) |  |  |
|  | CoDA  n=1  (Fairclough et al., 2021) |  |  | n=1  (Fairclough et al., 2021) |
|  | ISM  n=1  (Brown & Kwan, 2021) | Replacing ST with sleep or MVPA-> better self-esteem, n=1 (Brown & Kwan, 2021) |  |  |
|  | Latent profiles  n=1  (Brown, Cairney, et al., 2021) | High MVPA/low ST -> better self-esteem, n=1 (Brown, Cairney, et al., 2021) |  |  |
| Stress  n=3  (Bang et al., 2020; Monteagudo et al., 2023; Sampasa-Kanyinga et al., 2022a) | Meet guidelines yes/no  n=2  (Bang et al., 2020; Sampasa-Kanyinga et al., 2022a) | Meeting sleep -> lower stress, n=1 (Bang et al., 2020)  Meeting 3 guidelines -> lower stress n=1 (Sampasa-Kanyinga et al., 2022a)  Dose-response gradient n=1 (Sampasa-Kanyinga et al., 2022a) |  |  |
|  | ISM  n=1  (Monteagudo et al., 2023) | Replacing sedentary or LPA with MVPA -> lower stress n=1 (Monteagudo et al., 2023) |  |  |
| Quality of life/life satisfaction  n=4  (Dumuid et al., 2021; Janssen et al., 2017; Khan et al., 2021; Peralta et al., 2022) | Meet guidelines yes/no  n=3  (Janssen et al., 2017; Khan et al., 2021; Peralta et al., 2022) | Meeting 0 guidelines -> lower life satisfaction n=1 (Janssen et al., 2017), lower QoL, n=1 (Khan et al., 2021)  Meeting 3 guidelines -> higher life satisfaction n=1 (Peralta et al., 2022)  Dose-response gradient n=2 (Janssen et al., 2017; Khan et al., 2021) |  |  |
|  | CoDA  n=1  (Dumuid et al., 2021) | Higher sleep -> higher life satisfaction, n=1  Lower sedentary-> higher life satisfaction, n=1  Higher MVPA -> higher life satisfaction, n=1  (Dumuid et al., 2021) |  |  |
| Mental health/general distress/diagnosis  n=6  (Bang et al., 2020; Chong et al., 2021; Liang et al., 2023; Loewen et al., 2019; Sampasa-Kanyinga et al., 2022b; R. Zhang et al., 2023) | Meet guidelines yes/no  n=5  (Bang et al., 2020; Liang et al., 2023; Loewen et al., 2019; Sampasa-Kanyinga et al., 2022b; R. Zhang et al., 2023) | Meeting sleep -> better mental health, n=2 (Bang et al., 2020; R. Zhang et al., 2023)  Meeting ST -> better mental health, n=1 (Loewen et al., 2019; R. Zhang et al., 2023)  Meeting MVPA -> better mental health, n=2 (Loewen et al., 2019; R. Zhang et al., 2023)  Meeting MVPA + sleep, MVPA + ST -> better mental health n=1 (R. Zhang et al., 2023)  Meeting 0 guidelines -> worse mental health, n=2 (Liang et al., 2023; Sampasa-Kanyinga et al., 2022b)  Meeting 1 or 2 guidelines (not 3) -> better mental health n=1 (R. Zhang et al., 2023)  Dose-response gradient, n=3 (Liang et al., 2023; Loewen et al., 2019; Sampasa-Kanyinga et al., 2022b) |  |  |
|  | CoDA  n=1  (Chong et al., 2021) | Higher ST -> higher distress n=1  (Chong et al., 2021) |  |  |
| Flourishing  n= 6  (Brown, Cairney, et al., 2021; Brown & Kwan, 2021; Duncan et al., 2022, 2024; Faulkner et al., 2020; Gilchrist et al., 2021) | Meet guidelines yes/no  n=1  (Faulkner et al., 2020) | Meeting MVPA -> higher flourishing, n=1  Meeting sleep -> higher flourishing, n=1  (Faulkner et al., 2020) |  |  |
|  | CoDA  n=1 | Higher sleep -> higher flourishing n=1 (Duncan et al., 2024)  Higher MVPA -> higher flourishing n=1 (Duncan et al., 2024)  Lower ST -> higher flourishing n=1 (Duncan et al., 2024) |  |  |
|  | ISM  n=3  (Brown & Kwan, 2021; Duncan et al., 2022; Gilchrist et al., 2021) | Replacing ST with sleep -> better flourishing, n=3 (Brown & Kwan, 2021; Duncan et al., 2022; Gilchrist et al., 2021)  Replacing ST with MVPA -> better flourishing, n=2 (Brown & Kwan, 2021; Duncan et al., 2022)  Replacing sleep with MVPA-> better flourishing, n=2 (Brown & Kwan, 2021; Gilchrist et al., 2021) |  |  |
|  | Latent profiles  n=1  (Brown, Cairney, et al., 2021) | High MVPA/low ST -> better flourishing, n=1  Low MVPA/low ST-> better flourishing, n=1  (Brown, Cairney, et al., 2021) |  |  |
| Loneliness  n=1  (Burns et al., 2020) | Meet guidelines yes/no  n=1  (Burns et al., 2020) | Meeting 0 guidelines -> higher loneliness, n=1 (Burns et al., 2020) |  |  |

Table S4: findings summary adults (n=46)

| Outcome | Analyses | Positive associations with mental health | Negative associations with mental health | No significant finding |
| --- | --- | --- | --- | --- |
| Depression  n=29  (Blodgett et al., 2023; Brown, Hill, et al., 2022; Cabanas-Sánchez et al., 2021; Curtis et al., 2020; del Pozo Cruz et al., 2020; Dennis et al., 2021; Duncan et al., 2021; Feng et al., 2022; García-Hermoso et al., 2022; Haegele et al., 2021; Hofman et al., 2021; Jiang et al., 2024; Kandola et al., 2021; Kostick & Zhu, 2024; Larisch et al., 2020; K. Liang et al., 2021; W. Liang, Wang, Huang, et al., 2024; W. Liang, Wang, Su, et al., 2024; Lin et al., 2024; Liu et al., 2023, 2024; Meneguci et al., 2024; Murray et al., 2023; Ohta et al., 2023; Perez et al., 2021; Su et al., 2022; Wang et al., 2023; Y. Zhang et al., 2024; J. H. Zhu et al., 2024) | Meet guidelines yes/no  n=12  (Brown et al., 2022; Dennis et al., 2021; Feng et al., 2022; García-Hermoso et al., 2022; Haegele et al., 2021; Kostick & Zhu, 2024; K. Liang et al., 2021; W. Liang, Wang, Huang, et al., 2024; Lin et al., 2024; Ohta et al., 2023; Perez et al., 2021; Y. Zhang et al., 2024) | Meeting MVPA -> lower depression n=5 (Feng et al., 2022; García-Hermoso et al., 2022; Kostick & Zhu, 2024; K. Liang et al., 2021; W. Liang et al., 2024)  Meeting sedentary -> lower depression n=2 (Feng et al., 2022; K. Liang et al., 2021)  Meeting sleep -> lower depression n=6 (Brown et al., 2022; Haegele et al., 2021; K. Liang et al., 2021; W. Liang, Wang, Huang, et al., 2024; Lin et al., 2024; Perez et al., 2021)  Meeting ST -> lower depression n=4 (García-Hermoso et al., 2022; Kostick & Zhu, 2024; Lin et al., 2024; Perez et al., 2021)  Meeting MVPA+sedentary -> lower depression n=3 (Feng et al., 2022; K. Liang et al., 2021; W. Liang et al., 2024)  Meeting sedentary+sleep -> lower depression n=3 (Brown et al., 2022; Feng et al., 2022; Liang et al., 2021)  Meeting MVPA+sleep -> lower depression n=4 (Brown et al., 2022; K. Liang et al., 2021; W. Liang, Wang, Huang, et al., 2024; Lin et al., 2024)  Meeting ST + sleep -> lower depression n=1 (Lin et al., 2024)  Meeting 3 guidelines -> lower depression n=6 (Brown, Hill, et al., 2022; Feng et al., 2022; García-Hermoso et al., 2022; K. Liang et al., 2021; Lin et al., 2024; Perez et al., 2021)  Dose-response gradient n=7 (Dennis et al., 2021; Feng et al., 2022; Haegele et al., 2021; W. Liang et al., 2024; Ohta et al., 2023; Perez et al., 2021; Y. Zhang et al., 2024) |  |  |
|  | CoDA  n=11  (Blodgett et al., 2023; Cabanas-Sánchez et al., 2021; Curtis et al., 2020; del Pozo Cruz et al., 2020; Kandola et al., 2021; Larisch et al., 2020; W. Liang, Wang, Su, et al., 2024; Liu et al., 2024; Murray et al., 2023; Su et al., 2022; Wang et al., 2023) | Higher MVPA-> lower depression n=5 (Blodgett et al., 2023; Cabanas-Sánchez et al., 2021; W. Liang, Wang, Su, et al., 2024; Liu et al., 2024; Wang et al., 2023)  Higher sedentary -> higher depression n=5 (Blodgett et al., 2023; del Pozo Cruz et al., 2020; Kandola et al., 2021; W. Liang, Wang, Su, et al., 2024; Wang et al., 2023)  Higher LPA -> lower depression n=2 (Blodgett et al., 2023; W. Liang, Wang, Su, et al., 2024)  Higher sleep -> lower depression n=2 (W. Liang, Wang, Su, et al., 2024; Wang et al., 2023) | More sleep-> higher depression n=2 (Blodgett et al., 2023; Cabanas-Sánchez et al., 2021) | n=4  (Curtis et al., 2020; Larisch et al., 2020; Murray et al., 2023; Su et al., 2022) |
|  | ISM  n=15  (Blodgett et al., 2023; Cabanas-Sánchez et al., 2021; Curtis et al., 2020; del Pozo Cruz et al., 2020; Hofman et al., 2021; Kandola et al., 2021; Larisch et al., 2020; W. Liang, Wang, Su, et al., 2024; Liu et al., 2023, 2024; Meneguci et al., 2024; Murray et al., 2023; Su et al., 2022; Wang et al., 2023; J. H. Zhu et al., 2024) | Replacing sleep with MVPA -> lower depression n=4 (Blodgett et al., 2023; Cabanas-Sánchez et al., 2021; Hofman et al., 2021; Liu et al., 2024)  Replacing sleep with LPA -> lower depression n=1 (Blodgett et al., 2023)  Replacing sedentary with MVPA -> lower depression n=13 (Blodgett et al., 2023; Cabanas-Sánchez et al., 2021; del Pozo Cruz et al., 2020; Hofman et al., 2021; Kandola et al., 2021; W. Liang, Wang, Su, et al., 2024; Liu et al., 2023, 2024; Meneguci et al., 2024; Murray et al., 2023; Su et al., 2022; Wang et al., 2023; J. H. Zhu et al., 2024)  Replacing sedentary with LPA -> lower depression n=5 (Blodgett et al., 2023; Kandola et al., 2021; W. Liang, Wang, Su, et al., 2024; Liu et al., 2023; Su et al., 2022)  Replacing sedentary with sleep -> lower depression n=6 (Cabanas-Sánchez et al., 2021; del Pozo Cruz et al., 2020; W. Liang, Wang, Su, et al., 2024; Liu et al., 2023; Meneguci et al., 2024; Su et al., 2022)  Replacing LPA with MVPA -> lower depression n=4 (Blodgett et al., 2023; Cabanas-Sánchez et al., 2021; Liu et al., 2024; Wang et al., 2023)  Replacing LPA with sleep-> lower depression n=1 (Cabanas-Sánchez et al., 2021) | Replacing sedentary with LPA -> higher depression n=1 (Cabanas-Sánchez et al., 2021)  Replacing sedentary with sleep -> higher depression n=1 (Blodgett et al., 2023) | n=2  (Curtis et al., 2020; Larisch et al., 2020) |
|  | Latent profiles  n=1  (Jiang et al., 2024) | High MVPA/high sedentary/low sleep -> higher depression  High sedentary/high sleep -> higher depression  (Jiang et al., 2024) |  |  |
|  | SEM  n=1  (Duncan et al., 2021) | Higher composite activity-sleep score-> lower depression n=1 (Duncan et al., 2021) |  |  |
| Anxiety  n=13  (Bu et al., 2021; Chao et al., 2022; Curtis et al., 2020; Dennis et al., 2021; Duncan et al., 2021; Feng et al., 2022; Hofman et al., 2021; Kandola et al., 2021; Kostick & Zhu, 2024; Larisch et al., 2020; K. Liang et al., 2021; Lin et al., 2024; Wang et al., 2023) | Meet guidelines yes/no  n=6  (Bu et al., 2021; Dennis et al., 2021; Feng et al., 2022; Kostick & Zhu, 2024; K. Liang et al., 2021; Lin et al., 2024) | Meeting sedentary -> lower anxiety n=1 (Feng, Huang, et al., 2022)  Meeting sleep -> lower anxiety n=2 (K. Liang et al., 2021; Lin et al., 2024)  Meeting MVPA -> lower anxiety n=1 (K. Liang et al., 2021)  Meeting ST -> lower anxiety n=2 (Kostick & Zhu, 2024; Lin et al., 2024)  Meeting MVPA+sedentary -> lower anxiety n=1 (Feng et al., 2022; K. Liang et al., 2021)  Meeting sedentary+sleep -> lower anxiety n=2 (Feng et al., 2022; K. Liang et al., 2021)  Meeting MVPA+sleep -> lower anxiety n=1 (K. Liang et al., 2021)  Meeting ST + sleep -> lower anxiety n=1 (Lin et al., 2024)    Meeting 3 guidelines -> lower anxiety n=3 (Bu et al., 2021; K. Liang et al., 2021; Lin et al., 2024)  Dose-response gradient n=2 (Bu et al., 2021; Feng, Huang, et al., 2022) |  | n=1  (Dennis et al., 2021) |
|  | CoDA  n=4  (Curtis et al., 2020; Kandola et al., 2021; Larisch et al., 2020; Wang et al., 2023) | Higher sedentary -> higher anxiety n=2 (Kandola et al., 2021; Wang et al., 2023)  Higher MVPA -> lower anxiety n=1 (Wang et al., 2023)  Higher sleep -> lower anxiety n=1 (Wang et al., 2023) |  | n=2  (Curtis et al., 2020; Larisch et al., 2020) |
|  | ISM  n=6  (Chao et al., 2022; Curtis et al., 2020; Hofman et al., 2021; Kandola et al., 2021; Larisch et al., 2020; Wang et al., 2023) | Replacing sedentary with MVPA -> lower anxiety n=3 (Chao et al., 2022; Kandola et al., 2021; Wang et al., 2023)  Replacing sedentary with sleep -> lower anxiety n=1 (Kandola et al., 2021)  Replacing sedentary with LPA -> lower anxiety n=1 (Chao et al., 2022)  Replacing sleep with LPA -> lower anxiety n=1 (Chao et al., 2022)  Replacing sleep with MVPA -> lower anxiety n=1 (Chao et al., 2022)  Replacing LPA with MVPA -> lower anxiety n=2 (Chao et al., 2022; Wang et al., 2023) | Replacing sedentary with LPA -> higher anxiety n=1 (Kandola et al., 2021) | n=3  (Curtis et al., 2020; Hofman et al., 2021; Larisch et al., 2020) |
|  | SEM  n=1  (Duncan et al., 2021) | Higher composite activity-sleep score-> lower anxiety n=1 (Duncan et al., 2021) |  |  |
| Stress  n=7  (Curtis et al., 2020; Duncan et al., 2021; Feng et al., 2022; Kastelic et al., 2021; Larisch et al., 2020; Meyer et al., 2020; Wang et al., 2023) | Meet guidelines yes/no  n=2  (Feng, Huang, et al., 2022; Kastelic et al., 2021) | Meeting sedentary -> lower stress n=1 (Feng, Huang, et al., 2022)  Meeting sleep -> lower stress n=1 (Feng, Huang, et al., 2022)  Meeting MVPA+sedentary -> lower stress n=1 (Feng, Huang, et al., 2022)  Meeting sedentary+sleep -> lower stress n=1 (Feng, Huang, et al., 2022)  Meeting 0 guidelines -> higher stress n=1 (Kastelic et al., 2021)  Meeting 3 guidelines -> lower stress n=1 (Feng, Huang, et al., 2022)  Dose-response gradient n=2 (Feng, Huang, et al., 2022; Kastelic et al., 2021) |  |  |
|  | CoDA  n=3  (Curtis et al., 2020; Larisch et al., 2020; Wang et al., 2023) | Higher MVPA -> lower stress n=1 (Wang et al., 2023)  Higher sleep -> lower stress n=1 (Wang et al., 2023)  Higher sedentary -> higher stress n=1 (Wang et al., 2023) |  | n=2  (Curtis et al., 2020; Larisch et al., 2020) |
|  | ISM  n=4  (Curtis et al., 2020; Larisch et al., 2020; Meyer et al., 2020; Wang et al., 2023) | Replacing sedentary with sleep -> lower stress n=1 (Meyer et al., 2020)  Replacing sedentary with MVPA -> lower stress n=1 (Wang et al., 2023)  Replacing LPA with MVPA -> lower stress n=1 (Wang et al., 2023) |  | n=2  (Curtis et al., 2020; Larisch et al., 2020) |
|  | SEM  n=1  (Duncan et al., 2021) | Higher composite activity-sleep score-> lower stress n=1  (Duncan et al., 2021) |  |  |
| Quality of life/life satisfaction  n=7  (Cabanas-Sánchez et al., 2021; Curtis et al., 2020; Duncan et al., 2021; Guallar-Castillón et al., 2014; Hidde et al., 2022; Lin et al., 2024; Tabaczynski et al., 2020) | Meet guidelines yes/no  n=1  (Lin et al., 2024) | Meeting MVPA -> higher QoL n=1 (Lin et al., 2024)  Meeting ST -> higher QoL n=1 (Lin et al., 2024)  Meeting sleep -> higher QoL n=1 (Lin et al., 2024)  Meeting MVPA + sleep -> higher QoL n=1 (Lin et al., 2024)  Meeting ST + sleep -> higher QoL n=1 (Lin et al., 2024)  Meeting 3 guidelines -> higher QoL n=1 (Lin et al., 2024) |  |  |
|  | CoDA  n=2  (Cabanas-Sánchez et al., 2021; Curtis et al., 2020) | Higher MVPA -> higher happiness n=1 (Cabanas-Sánchez et al., 2021) |  | n=1  (Curtis et al., 2020) |
|  | ISM  n=4  (Cabanas-Sánchez et al., 2021; Curtis et al., 2020; Hidde et al., 2022; Tabaczynski et al., 2020) | Replacing sleep with MVPA -> higher happiness n=1 (Cabanas-Sánchez et al., 2021)  Replacing sedentary with MVPA -> higher happiness n=1 (Cabanas-Sánchez et al., 2021), higher QoL n=1 (Tabaczynski et al., 2020)  Replacing sedentary with sleep -> higher QoL n=1 (Tabaczynski et al., 2020)  Replacing LPA with MVPA -> higher happiness n=1 (Cabanas-Sánchez et al., 2021) |  | n=2  (Curtis et al., 2020; Hidde et al., 2022) |
|  | Other  SEM: n=1 (Duncan et al., 2021)  PCA: n=1 (Guallar-Castillón et al., 2014) | Higher composite activity-sleep score-> higher QoL n=1 (Duncan et al., 2021)  High MVPA+high sedentary -> better QoL n=1 (Guallar-Castillón et al., 2014)  High LPA+high sedentary -> better QoL n=1 (Guallar-Castillón et al., 2014) |  |  |
| Mental health/well-being/mood/general distress  n=14  (Baillot et al., 2022; Brown, Faulkner, et al., 2022; Cabanas-Sánchez et al., 2021; Colley et al., 2018; Hajo et al., 2020; Kitano et al., 2020; Larisch et al., 2020; Le et al., 2021; Luo et al., 2022; McGregor et al., 2018; Meyer et al., 2020; Murray et al., 2023; Perez et al., 2021; Weatherson et al., 2021) | Meet guidelines yes/no  n=5  (Baillot et al., 2022; Hajo et al., 2020; Luo et al., 2022; Perez et al., 2021; Weatherson et al., 2021) | Meeting MVPA -> better mental health n=2 (Hajo et al., 2020; Luo et al., 2022), lower distress n=1 (Perez et al., 2021)  Meeting sedentary -> better mood n=1 (Hajo et al., 2020)  Meeting sleep -> better mental health n=2 (Baillot et al., 2022; Perez et al., 2021)  Meeting ST -> better mental health n=1 (Luo et al., 2022)  Meeting MVPA + sleep -> better mental health n=1 (Luo et al., 2022)  Meeting 3 guidelines -> better mood n=1 (Hajo et al., 2020), better mental health n=1 (Weatherson et al., 2021)  Dose-response gradient n=2 (Luo et al., 2022; Perez et al., 2021) |  |  |
|  | CoDA  n=6  (Cabanas-Sánchez et al., 2021; Kitano et al., 2020; Larisch et al., 2020; Le et al., 2021; McGregor et al., 2018; Murray et al., 2023) | Higher sleep -> lower distress n=1 (Kitano et al., 2020)  Higher sedentary -> higher distress n=1 (Kitano et al., 2020)  Higher MVPA -> higher wellbeing n=1 (Larisch et al., 2020), higher positive affect n=1 (Le et al., 2021), higher mental health n=3 (Cabanas-Sánchez et al., 2021; McGregor et al., 2018; Murray et al., 2023) | Higher LPA -> higher distress n=1 (Kitano et al., 2020) |  |
|  | ISM  n=7  (Cabanas-Sánchez et al., 2021; Colley et al., 2018; Kitano et al., 2020; Larisch et al., 2020; Le et al., 2021; Meyer et al., 2020; Murray et al., 2023) | Replacing sedentary with LPA -> better mental health n=1 (Colley et al., 2018), better mood n=1 (Meyer et al., 2020)  Replacing sedentary with MVPA -> better mental health n=2 (Larisch et al., 2020; Murray et al., 2023), better mood n=2 (Le et al., 2021; Meyer et al., 2020)  Replacing sedentary with sleep -> lower distress n=1 (Kitano et al., 2020), better mood n=1 (Meyer et al., 2020), better mental health n=1 (Colley et al., 2018)  Replacing LPA with sleep -> lower distress n=1 (Kitano et al., 2020)  Replacing LPA with MVPA -> higher mental health n=2 (Larisch et al., 2020; Murray et al., 2023), better mood n=1 (Le et al., 2021)  Replacing sleep with MVPA -> higher wellbeing n=1 (Larisch et al., 2020), better mood n=1 (Le et al., 2021), better mental health n=2 (Cabanas-Sánchez et al., 2021; Murray et al., 2023) |  |  |
|  | Latent profiles  n=1  (Brown, Faulkner, et al., 2022) | High MVPA/low ST -> higher wellbeing, lower distress n=1  (Brown, Faulkner, et al., 2022) |  |  |
| Loneliness  n=3  (Cabanas-Sánchez et al., 2021; W. Liang, Wang, Huang, et al., 2024; W. Liang, Wang, Su, et al., 2024) | Meet guidelines yes/no  n=1  (W. Liang, Wang, Huang, et al., 2024) | Dose-response gradient n=1 (W. Liang, Wang, Huang, et al., 2024) |  |  |
|  | CoDA  n=2  (Cabanas-Sánchez et al., 2021; W. Liang, Wang, Su, et al., 2024) | Higher MVPA -> lower loneliness n=2 (Cabanas-Sánchez et al., 2021; W. Liang, Wang, Su, et al., 2024)  Higher LPA -> lower loneliness n=1 (W. Liang, Wang, Su, et al., 2024)  Higher sleep -> lower loneliness n=1 (W. Liang, Wang, Su, et al., 2024)  Higher sedentary -> higher loneliness n=1 (W. Liang, Wang, Su, et al., 2024) |  |  |
|  | ISM  n=2  (Cabanas-Sánchez et al., 2021; W. Liang, Wang, Su, et al., 2024) | Replacing sleep with MVPA -> lower loneliness n=1  Replacing sedentary with MVPA -> lower loneliness n=2 (Cabanas-Sánchez et al., 2021; W. Liang, Wang, Su, et al., 2024)  Replacing sedentary with LPA -> lower loneliness n=1 (W. Liang, Wang, Su, et al., 2024)  Replacing sedentary with sleep -> lower loneliness n=1 (W. Liang, Wang, Su, et al., 2024)  Replacing LPA with MVPA -> lower loneliness n=1  (Cabanas-Sánchez et al., 2021) |  |  |
| Work-related mental health  n=3  (Hajo et al., 2020; Kitano et al., 2020; Larisch et al., 2020) | Meet guidelines yes/no  n=1  (Hajo et al., 2020) |  |  | n=1  (Hajo et al., 2020) |
|  | CoDA  n=2  (Kitano et al., 2020; Larisch et al., 2020) | Higher sleep -> higher work engagement n=1  Higher sedentary -> lower work engagement n=1  (Kitano et al., 2020) |  | n=1  (Larisch et al., 2020) |
|  | ISM  n=2  (Kitano et al., 2020; Larisch et al., 2020) |  |  | n=2  (Kitano et al., 2020; Larisch et al., 2020) |
| PTSD  n=2  (Feng, Lau, et al., 2022; Perez et al., 2021) | Meet guidelines yes/no  n=2  (Feng, Lau, et al., 2022; Perez et al., 2021) | Meeting sleep -> less PTSD n=2 (Feng, Lau, et al., 2022; Perez et al., 2021)  Meeting sedentary -> less PTSD n=1 (Feng, Lau, et al., 2022)  Dose-response gradient n=1  (Perez et al., 2021) | Meeting MVPA -> more PTSD n=1 (Feng, Lau, et al., 2022) |  |

References

Bang, F., Roberts, K. C., Chaput, J.-P., Goldfield, G. S., & Prince, S. A. (2020). Physical activity, screen time and sleep duration: Combined associations with psychosocial health among Canadian children and youth. *Health Reports*, *31*(5), 9–16. https://doi.org/10.25318/82-003-x202000500002-eng

Bao, R., Yang, Z., Memon, A. R., Chen, S., Wang, L., & Cai, Y. (2024). Association between meeting the 24‐h movement guidelines and psychosocial health in children: A cross‐sectional study. *Child: Care, Health and Development*, *50*(1), e13191. https://doi.org/10.1111/cch.13191

Blodgett, J. M., Mitchell, J. J., Stamatakis, E., Chastin, S., & Hamer, M. (2023). Associations between the composition of daily time spent in physical activity, sedentary behaviour and sleep and risk of depression: Compositional data analyses of the 1970 British cohort Study. *Journal of Affective Disorders*, *320*, 616–620. https://doi.org/10.1016/j.jad.2022.09.110

Brown, D. M. Y., Cairney, J., & Kwan, M. Y. (2021). Adolescent movement behaviour profiles are associated with indicators of mental wellbeing. *Mental Health and Physical Activity*, *20*, 100387. https://doi.org/10.1016/J.MHPA.2021.100387

Brown, D. M. Y., Faulkner, G. E. J., & Kwan, M. Y. W. (2022). Healthier movement behavior profiles are associated with higher psychological wellbeing among emerging adults attending post-secondary education. *Journal of Affective Disorders*, *319*, 511–517. https://doi.org/10.1016/j.jad.2022.09.111

Brown, D. M. Y., Hill, R. M., & Wolf, J. K. (2022). Cross-sectional associations between 24-h movement guideline adherence and suicidal thoughts among Canadian post-secondary students. *Mental Health and Physical Activity*, *23*, 100484. https://doi.org/10.1016/j.mhpa.2022.100484

Brown, D. M. Y., Kwan, M. Y., Arbour-Nicitopoulos, K. P., & Cairney, J. (2021). Identifying patterns of movement behaviours in relation to depressive symptoms during adolescence: A latent profile analysis approach. *Preventive Medicine*, *143*, 106352. https://doi.org/10.1016/J.YPMED.2020.106352

Brown, D. M. Y., & Kwan, M. Y. W. (2021). Movement Behaviors and Mental Wellbeing: A Cross-Sectional Isotemporal Substitution Analysis of Canadian Adolescents. *Frontiers in Behavioral Neuroscience*, *15*. https://doi.org/10.3389/fnbeh.2021.736587

Brown, D. M. Y., Kwan, M. Y. W., King-Dowling, S., & Cairney, J. (2021). Cross-Sectional Associations Between Wake-Time Movement Compositions and Mental Health in Preschool Children With and Without Motor Coordination Problems. *Frontiers in Pediatrics*, *9*, 752333. https://doi.org/10.3389/FPED.2021.752333/BIBTEX

Bu, H., He, A., Gong, N., Huang, L., Liang, K., Kastelic, K., Ma, J., Liu, Y., Chen, S. T., & Chi, X. (2021). Optimal movement behaviors: correlates and associations with anxiety symptoms among Chinese university students. *BMC Public Health*, *21*(1), 2052. https://doi.org/10.1186/S12889-021-12116-6/FIGURES/1

Burns, R. D., Bai, Y., Pfledderer, C. D., Brusseau, T. A., & Byun, W. (2020). Movement behaviors and perceived loneliness and sadness within alaskan adolescents. *International Journal of Environmental Research and Public Health*, *17*(18), 1–13. https://doi.org/10.3390/IJERPH17186866/IJERPH_17_06866_PDF.PDF

Cabanas-Sánchez, V., Esteban-Cornejo, I., García-Esquinas, E., Ortolá, R., Ara, I., Rodríguez-Gómez, I., Chastin, S. F. M., Rodríguez-Artalejo, F., & Martínez-Gómez, D. (2021). Cross-sectional and prospective associations of sleep, sedentary and active behaviors with mental health in older people: a compositional data analysis from the Seniors-ENRICA-2 study. *International Journal of Behavioral Nutrition and Physical Activity*, *18*(1). https://doi.org/10.1186/s12966-021-01194-9

Cao, R., Gao, T., Hu, Y., Qin, Z., Ren, H., Liang, L., Li, C., & Mei, S. (2020). Clustering of lifestyle factors and the relationship with depressive symptoms among adolescents in Northeastern China. *Journal of Affective Disorders*, *274*, 704–710. https://doi.org/10.1016/j.jad.2020.05.064

Carson, V., Ezeugwu, V. E., Tamana, S. K., Chikuma, J., Lefebvre, D. L., Azad, M. B., Moraes, T. J., Subbarao, P., Becker, A. B., Turvey, S. E., Sears, M. R., & Mandhane, P. J. (2019). Associations between meeting the Canadian 24-Hour Movement Guidelines for the Early Years and behavioral and emotional problems among 3-year-olds. *Journal of Science and Medicine in Sport*, *22*, 797–802. https://doi.org/10.1016/j.jsams.2019.01.003

Chao, L., Ma, R., & Jiang, W. (2022). Movement behaviours and anxiety symptoms in Chinese college students: A compositional data analysis. *Frontiers in Psychology*, *13*, 952728. https://doi.org/10.3389/FPSYG.2022.952728/BIBTEX

Chong, K. H., Parrish, A.-M., Cliff, D. P., Dumuid, D., & Okely, A. D. (2021). Cross-sectional and longitudinal associations between 24-hour movement behaviours, recreational screen use and psychosocial health outcomes in children: A compositional data analysis approach. *International Journal of Environmental Research and Public Health*, *18*(11). https://doi.org/10.3390/ijerph18115995

Colley, R. C., Michaud, I., & Garriguet, D. (2018). Reallocating time between sleep, sedentary and active behaviours: Associations with obesity and health in Canadian adults. *Health Reports*, *29*(4), 3–13. www.statcan.gc.ca

Curtis, R. G., Dumuid, D., Olds, T., Plotnikoff, R., Vandelanotte, C., Ryan, J., Edney, S., & Maher, C. (2020). The Association Between Time-Use Behaviors and Physical and Mental Well-Being in Adults: A Compositional Isotemporal Substitution Analysis. *Journal of Physical Activity and Health*, *17*(2), 197–203. https://doi.org/10.1123/JPAH.2018-0687

de Faria, F. R., Barbosa, D., Howe, C. A., Canabrava, K. L. R., Sasaki, J. E., & dos Santos Amorim, P. R. (2022). Time-use movement behaviors are associated with scores of depression/anxiety among adolescents: A compositional data analysis. *PLOS ONE*, *17*(12), e0279401. https://doi.org/10.1371/journal.pone.0279401

del Pozo Cruz, B., Alfonso-Rosa, R. M., Mcgregor, D., Chastin, S. F. M., Palarea-Albaladejo, J., & del Pozo Cruz, J. (2020). Sedentary behaviour is associated with depression symptoms: Compositional data analysis from a representative sample of 3233 US adults and older adults assessed with accelerometers. *Journal of Affective Disorders*, *265*(April 2019), 59–62. https://doi.org/10.1016/j.jad.2020.01.023

Dennis, C.-L., Birken, C. S., Brennenstuhl, S., Wrottesley, S., Marini, F., Bell, R. C., Tremblay, M. S., & Prioreschi, A. (2021). Describing 24-hour movement behaviours among preconception and recently pregnant Canadian parents: who do we need to target? *Behavioral Medicine*. https://doi.org/10.1080/08964289.2021.1987854

Dumuid, D., Olds, T., Lange, K., Edwards, B., Lycett, K., Burgner, D. P., Simm, P., Dwyer, T., Le, H., & Wake, M. (2021). Goldilocks Days: optimising children’s time use for health and well-being. *Journal of Epidemiology and Community Health*, *76*(3), 301–308. https://doi.org/10.1136/jech-2021-216686

Duncan, M. J., Kuzik, N., Silva, D. A. S., Bélanger, R. E., Carson, V., Chaput, J.-P., Faulkner, G., Ferro, M. A., Turcotte-Tremblay, A.-M., Leatherdale, S. T., Patte, K. A., & Tremblay, M. S. (2024). “Goldilocks days” for adolescent mental health: Movement behaviour combinations for well-being, anxiety and depression by gender. *Mental Health and Physical Activity*, *26*, 100572. https://doi.org/10.1016/j.mhpa.2023.100572

Duncan, M. J., Rayward, A. T., Holliday, E. G., Brown, W. J., Vandelanotte, C., Murawski, B., & Plotnikoff, R. C. (2021). Effect of a physical activity and sleep m-health intervention on a composite activity-sleep behaviour score and mental health: a mediation analysis of two randomised controlled trials. *International Journal of Behavioral Nutrition and Physical Activity*, *18*(1). https://doi.org/10.1186/s12966-021-01112-z

Duncan, M. J., Riazi, N. A., Faulkner, G., Gilchrist, J. D., Leatherdale, S. T., & Patte, K. A. (2022). The association of physical activity, sleep, and screen time with mental health in Canadian adolescents during the COVID-19 pandemic: A longitudinal isotemporal substitution analysis. *Mental Health and Physical Activity*, *23*, 100473. https://doi.org/10.1016/j.mhpa.2022.100473

Fairclough, S. J., Clifford, L., Brown, D., & Tyler, R. (2023). Characteristics of 24-hour movement behaviours and their associations with mental  health in children and adolescents. *Journal of Activity, Sedentary and Sleep Behaviors*, *2*(1), 11. https://doi.org/10.1186/s44167-023-00021-9

Fairclough, S. J., Tyler, R., Dainty, J. R., Dumuid, D., Richardson, C., Shepstone, L., & Atkin, A. J. (2021). Cross-sectional associations between 24-hour activity behaviours and mental health indicators in children and adolescents: A compositional data analysis. *Journal of Sports Sciences*, *39*(14), 1602–1614. https://doi.org/10.1080/02640414.2021.1890351

Faulkner, G., Weatherson, K., Patte, K., Qian, W., & Leatherdale, S. T. (2020). Are one-year changes in adherence to the 24-hour movement guidelines associated with flourishing among Canadian youth? *Preventive Medicine*, *139*, 106179. https://doi.org/10.1016/J.YPMED.2020.106179

Feng, J., Huang, W. Y., Lau, P. W. C., Wong, S. H.-S., & Sit, C. H.-P. (2022). Movement behaviors and mental health of caregivers of preschoolers in China during the COVID-19 pandemic. *Preventive Medicine*, *155*, 106913. https://doi.org/10.1016/j.ypmed.2021.106913

Feng, J., Lau, P. W. C., Shi, L., & Huang, W. Y. (2022). Longitudinal Shifts in Movement Behaviors during the COVID-19 Pandemic: Relations  to Posttraumatic Stress Disorder among University Students. *International Journal of Environmental Research and Public Health*, *19*(20). https://doi.org/10.3390/ijerph192013449

Fung, H., Yeo, B. T. T., Chen, C., Lo, J. C., Chee, M. W. L., & Ong, J. L. (2023). Adherence to 24-Hour Movement Recommendations and Health Indicators in Early Adolescence: Cross-Sectional and Longitudinal Associations in the Adolescent Brain Cognitive Development Study. *Journal of Adolescent Health*, *72*(3), 460–470. https://doi.org/10.1016/j.jadohealth.2022.10.019

García-Hermoso, A., Ezzatvar, Y., Ramírez-Vélez, R., López-Gil, J. F., & Izquierdo, M. (2022). Trajectories of 24-h movement guidelines from middle adolescence to adulthood on  depression and suicidal ideation: a 22-year follow-up study. *The International Journal of Behavioral Nutrition and Physical Activity*, *19*(1), 135. https://doi.org/10.1186/s12966-022-01367-0

Gilchrist, J. D., Battista, K., Patte, K. A., Faulkner, G., Carson, V., & Leatherdale, S. T. (2021). Effects of reallocating physical activity, sedentary behaviors, and sleep on mental health in adolescents. *Mental Health and Physical Activity*, *20*, 100380. https://doi.org/10.1016/J.MHPA.2020.100380

Guallar-Castillón, P., Bayán-Bravo, A., León-Muñoz, L. M., Balboa-Castillo, T., López-García, E., Gutierrez-Fisac, J. L., & Rodríguez-Artalejo, F. (2014). The association of major patterns of physical activity, sedentary behavior and sleep with health-related quality of life: A cohort study. *Preventive Medicine*, *67*, 248–254. https://doi.org/10.1016/J.YPMED.2014.08.015

Haegele, J. A., Zhu, X., & Healy, S. (2021). Behavioral Correlates of Depression Among Adults with Visual Impairments. *Journal of Visual Impairment and Blindness*. https://doi.org/10.1177/0145482X211046696

Hajo, S., Reed, J. L., Hans, H., Tulloch, H. E., Reid, R. D., & Prince, S. A. (2020). Physical activity, sedentary time and sleep and associations with mood states, shift work disorder and absenteeism among nurses: An analysis of the cross-sectional Champlain Nurses’ Study. *PeerJ*, *8*, e8464. https://doi.org/10.7717/PEERJ.8464/PEERJ_08_8464_PDF.PDF

Hansen, J., Hanewinkel, R., & Galimov, A. (2022). Physical activity, screen time, and sleep: do German children and adolescents meet the movement guidelines? *European Journal of Pediatrics*, *181*(5), 1985–1995. https://doi.org/10.1007/S00431-022-04401-2/TABLES/3

Hinkley, T., Timperio, A., Watson, A., Duckham, R. L., Okely, A. D., Cliff, D., Carver, A., & Hesketh, K. D. (2020). Prospective associations with physiological, psychosocial and educational outcomes of meeting Australian 24-Hour Movement Guidelines for the Early Years. *International Journal of Behavioral Nutrition and Physical Activity*, *17*(36), 1–12. https://doi.org/10.1186/S12966-020-00935-6/TABLES/3

Hofman, A., Voortman, T., Ikram, M. A., & Luik, A. I. (2021). Substitutions of physical activity, sedentary behaviour and sleep: associations with mental health in middle-aged and elderly persons. *J Epidemiol Community Health*, *76*(2), 175–181. https://doi.org/10.1136/JECH-2020-215883

Hou, M., Herold, F., Healy, S., Haegele, J. A., Block, M. E., Ludyga, S., Schuch, F. B., Ng, J. L., Gerber, M., Hossain, M. M., Taylor, A., Van Damme, T., Müller, N. G., Kramer, A. F., & Zou, L. (2023). 24-Hour movement behaviors among visually impaired US children and adolescents. *Mental Health and Physical Activity*, *25*. https://doi.org/10.1016/j.mhpa.2023.100545

Hou, M., Herold, F., Werneck, A. O., Teychenne, M., Paoli, A. G. D., Taylor, A., Van Damme, T., Kramer, A. F., Hossain, M. M., Yeung, A. S., Owen, N., Gerber, M., Ludyga, S., Cheval, B., & Zou, L. (2024). Associations of 24-hour movement behaviors with externalizing and internalizing problems among children and adolescents prescribed with eyeglasses/contact lenses. *International Journal of Clinical and Health Psychology*, *24*(1), 100435. https://doi.org/10.1016/J.IJCHP.2023.100435

Huang, C., Yang, Y., Lu, C., Qin, P., Jiang, W., Ma, J., & Guo, L. (2024). Associations of 24-hour movement behaviors with emotional and behavioral problems among Chinese adolescents. *Social Psychiatry and Psychiatric Epidemiology*, 1–13. https://doi.org/10.1007/S00127-024-02712-3/TABLES/4

Janssen, I., Roberts, K. C., & Thompson, W. (2017). Is adherence to the Canadian 24-hour movement behaviour guidelines for school-aged children and youth associated with improved indicators of physical, mental, and social health? *Applied Physiology, Nutrition and Metabolism*, *42*, 725–731. https://doi.org/10.1139/apnm-2016-0681

Jiang, Y., Lu, Y., Cui, J., & Chu, A. (2024). 24-hour movement behaviors time allocation and depression among Chinese community-dwelling older adults: A latent profile analysis. *Geriatric Nursing*, *58*, 382–387. https://doi.org/10.1016/j.gerinurse.2024.05.007

Kandola, A. A., del Pozo Cruz, B., Osborn, D. P. J., Stubbs, B., Choi, K. W., & Hayes, J. F. (2021). Impact of replacing sedentary behaviour with other movement behaviours on depression and anxiety symptoms: a prospective cohort study in the UK Biobank. *BMC Medicine*, *19*(133). https://doi.org/10.1186/S12916-021-02007-3

Kasai, A., Shikano, A., Tanaka, R., Yoshinaga, M., & Noi, S. (2024). School-aged children’s movement behaviours and subjective health complaints in  Japan: a cross-sectional study during COVID-19 pandemic-related school closures and after school reopenings. *BMC Public Health*, *24*(1), 1204. https://doi.org/10.1186/s12889-024-18712-6

Kastelic, K., Pedišić, Ž., Lipovac, D., Kastelic, N., Chen, S. T., & Šarabon, N. (2021). Associations of meeting 24-h movement guidelines with stress and self-rated health among adults: is meeting more guidelines associated with greater benefits? *BMC Public Health*, *21*, 929. https://doi.org/10.1186/S12889-021-10979-3/TABLES/3

Khan, A., Ahmed, K. R., & Lee, E. Y. (2024). Adherence to 24-hour movement guidelines and their association with depressive symptoms in adolescents: Evidence from Bangladesh. *Sports Medicine and Health Science*, *6*(1), 76–81. https://doi.org/10.1016/J.SMHS.2023.10.003

Khan, A., Lee, E. Y., & Tremblay, M. S. (2021). Meeting 24-h movement guidelines and associations with health related quality of life of Australian adolescents. *Journal of Science and Medicine in Sport*, *24*(5), 468–473. https://doi.org/10.1016/J.JSAMS.2020.10.017

Kitano, N., Kai, Y., Jindo, T., Tsunoda, K., & Arao, T. (2020). Compositional data analysis of 24-hour movement behaviors and mental health in workers. *Preventive Medicine Reports*, *20*. https://doi.org/10.1016/j.pmedr.2020.101213

Kostick, M. D., & Zhu, X. (2024). Movement Behaviors and Mental Health of Catholic Priests in the Eastern United States. *Journal of Religion and Health*, *63*(3), 1867–1879. https://doi.org/10.1007/S10943-023-01894-5/TABLES/3

Kuzik, N., Naylor, P.-J., Spence, J. C., & Carson Id, V. (2020). Movement behaviours and physical, cognitive, and social-emotional development in preschool-aged children: Cross-sectional associations using compositional analyses. *PlosOne*, *15*(8), e0237945. https://doi.org/10.1371/journal.pone.0237945

Larisch, L.-M., Kallings, L. v, Hagströmer, M., Desai, M., Rosen, P. v, & Blom, V. (2020). Associations between 24 h movement behavior and mental health in office workers. *International Journal of Environmental Research and Public Health*, *17*(17), 1–20. https://doi.org/10.3390/ijerph17176214

Le, F., Yap, Y., Yan, N., Tung, C., Bei Bei, ·, & Wiley, J. F. (2021). The Associations Between Daily Activities and Affect: a Compositional Isotemporal Substitution Analysis. *International Journal of Behavioral Medicine*, *3*. https://doi.org/10.1007/s12529-021-10031-z

Li, F., Yin, L., Luo, W., Gao, Z., Ryu, S., Sun, M., Liu, P., & Yang, Z. (2024). Isotemporal substitution effect of 24-hour movement behavior on the mental health of Chinese preschool children. *Frontiers in Public Health*, *12*, 1288262. https://doi.org/10.3389/FPUBH.2024.1288262/BIBTEX

Liang, K., Chen, S., & Chi, X. (2023). Differential Associations Between Meeting 24-Hour Movement Guidelines With Mental Wellbeing and Mental Illness Among Chinese Adolescents. *Journal of Adolescent Health*, *72*(5), 658–666. https://doi.org/10.1016/j.jadohealth.2022.11.231

Liang, K., de Lucena Martins, C. M., Chen, S.-T., Clark, C. C. T., Duncan, M. J., Bu, H., Huang, L., & Chi, X. (2021). Sleep as a priority: 24-hour movement guidelines and mental health of chinese college students during the covid-19 pandemic. *Healthcare (Switzerland)*, *9*(9). https://doi.org/10.3390/healthcare9091166

Liang, W., Wang, Y., Huang, Q., Shang, B., Su, N., Zhou, L., Rhodes, R. E., Steven Baker, J., & Duan, Y. (2024). Adherence to 24-Hour Movement Guidelines Among Chinese Older Adults: Prevalence, Correlates, and Associations With Physical and Mental Health Outcomes. *JMIR Public Health and Surveillance*, *10*, e46072. https://doi.org/10.2196/46072

Liang, W., Wang, Y., Su, N., Song, H., Rhodes, R. E., Wang, X., Shang, B., Zhou, L., Huang, Q., Bu, D., Baker, J. S., & Duan, Y. (2024). Associations of Reallocating Sedentary Time to Physical Activity and Sleep with Physical and Mental Health of Older Adults. *Medicine & Science in Sports & Exercise*, *56*(10), 1935–1944. https://doi.org/10.1249/MSS.0000000000003491

Lin, L., Liang, W., Wang, R., Rhodes, R. E., & Liu, H. (2024). Association of 24-hour movement guideline adherence, mental health and quality of life in young adults: the role of e-Health literacy. *Frontiers in Public Health*, *12*, 1344718. https://doi.org/10.3389/FPUBH.2024.1344718/BIBTEX

Liu, Y., Ge, X., Wang, Y., Qiao, S., & Cai, Y. (2024). How race and socioeconomic status moderate the association between moderate-to-vigorous physical activity and depressive symptoms: a cross-sectional study with compositional data. *British Journal of Sports Medicine*, bjsports-2024-108290. https://doi.org/10.1136/bjsports-2024-108290

Liu, Y., Lin, H., Zhang, H., Zhang, X., & Yin, S. (2023). Correlation analysis between physical activity and depressive tendencies among  occupational groups: an isotemporal substitution approach. *BMC Public Health*, *23*(1), 2241. https://doi.org/10.1186/s12889-023-17134-0

Loewen, O. K., Maximova, K., Ekwaru, J. P., Faught, E. L., Asbridge, M., Ohinmaa, A., & Veugelers, P. J. (2019). Lifestyle behavior and mental health in early adolescence. *Pediatrics*, *143*(5), e20183307. https://doi.org/10.1542/PEDS.2018-3307

López-Gil, J. F., Firth, J., & García-Hermoso, A. (2024). Is meeting with the 24-h movement recommendations linked with suicidality? Results from a nationwide sample of 44,734 U.S. adolescents. *Journal of Affective Disorders*, *349*, 617–624. https://doi.org/10.1016/J.JAD.2024.01.019

López-Gil, J. F., Roman-Viñas, B., Aznar, S., & Tremblay, M. S. (2022). Meeting 24-h movement guidelines: Prevalence, correlates, and associations with socioemotional behavior in Spanish minors. *Scandinavian Journal of Medicine & Science in Sports*, *32*(5), 881–891. https://doi.org/10.1111/SMS.14132

Lu, S., Cheval, B., Yu, Q., Hossain, M. M., Chen, S. T., Taylor, A., Bao, R., Doig, S., Li, J., Wang, T., Yan, Z., Kuang, J., Jiao, C., & Zou, L. (2021). Associations of 24-Hour Movement Behavior with Depressive Symptoms and Anxiety in Children: Cross-Sectional Findings from a Chinese Sample. *Healthcare*, *9*, 1532. https://doi.org/10.3390/HEALTHCARE9111532

Luo, L., Cao, Y., Hu, Y., Wen, S., Tang, K., Ding, L., & Song, N. (2022). The Associations between Meeting 24-Hour Movement Guidelines (24-HMG) and  Self-Rated Physical and Mental Health in Older Adults-Cross Sectional Evidence from China. *International Journal of Environmental Research and Public Health*, *19*(20). https://doi.org/10.3390/ijerph192013407

Luo, L., Zeng, X., Cao, Y., Hu, Y., Wen, S., Tang, K., Ding, L., Wang, X., & Song, N. (2023). The Associations between Meeting 24-Hour Movement Guidelines (24-HMG) and Mental  Health in Adolescents-Cross Sectional Evidence from China. *International Journal of Environmental Research and Public Health*, *20*(4). https://doi.org/10.3390/ijerph20043167

McGregor, D., Carson, V., Palarea-Albaladejo, J., Dall, P., Tremblay, M., & Chastin, S. (2018). Compositional Analysis of the Associations between 24-h Movement Behaviours and Health Indicators among Adults and Older Adults from the Canadian Health Measure Survey. *International Journal of Environmental Research and Public Health*, *15*(8), 1779. https://doi.org/10.3390/ijerph15081779

Mcneill, J., Howard, S. J., Vella, S. A., & Cliff, D. P. (2020). Compliance with the 24-Hour movement guidelines for the early years: Cross-sectional and longitudinal associations with executive function and psychosocial health in preschool children. *Journal of Science and Medicine in Sport*, *23*, 846–853. https://doi.org/10.1016/j.jsams.2020.02.011

Meneguci, J., Galvão, L. L., Tribess, S., Meneguci, C. A. G., & Virtuoso Júnior, J. S. (2024). Isotemporal substitution analysis of time between sleep, sedentary behavior, and  physical activity on depressive symptoms in older adults: a cross-sectional study. *Sao Paulo Medical Journal = Revista Paulista de Medicina*, *142*(4), e2023144. https://doi.org/10.1590/1516-3180.2023.0144.R2.04122023

Meyer, J. D., Ellingson, L. D., Buman, M. P., Shook, R. P., Hand, G. A., & Blair, S. N. (2020). Current and 1-Year Psychological and Physical Effects of Replacing Sedentary Time With Time in Other Behaviors. *American Journal of Preventive Medicine*, *59*(1), 12–20. https://doi.org/10.1016/j.amepre.2020.02.018

Monteagudo, P., Beltran-Valls, M. R., Adelantado-Renau, M., & Moliner-Urdiales, D. (2023). Observational longitudinal association between waking movement behaviours and psychological distress among adolescents using isotemporal analysis: DADOS study. *Journal of Sports Sciences*, *41*(13), 1290–1298. https://doi.org/10.1080/02640414.2023.2268359

Murray, R. M., Doré, I., Sabiston, C. M., Michael, F., & O’Loughlin, J. L. (2023). A time compositional analysis of the association between movement behaviors and indicators of mental health in young adults. *Scandinavian Journal of Medicine & Science in Sports*, *33*(12), 2598–2607. https://doi.org/10.1111/sms.14471

Ohta, T., Ogawa, M., Kikuchi, N., Sasai, H., & Okamoto, T. (2023). Adherence to 24-h Movement Guidelines and Depressive Status During the Coronavirus Disease Outbreak: A Cross-Sectional Japanese Survey. *International Journal of Public Health*, *68*, 1604647. https://doi.org/10.3389/IJPH.2023.1604647/BIBTEX

Patte, K. A., Faulkner, G., Qian, W., Duncan, M., & Leatherdale, S. T. (2020). Are one-year changes in adherence to the 24-hour movement guidelines associated with depressive symptoms among youth? *BMC Public Health*, *20*(1), 793. https://doi.org/10.1186/S12889-020-08887-Z/TABLES/3

Peralta, G. P., Camerini, A. L., Haile, S. R., Kahlert, C. R., Lorthe, E., Marciano, L., Nussbaumer, A., Radtke, T., Ulyte, A., Puhan, M. A., & Kriemler, S. (2022). Lifestyle Behaviours of Children and Adolescents During the First Two Waves of the COVID-19 Pandemic in Switzerland and Their Relation to Well-Being: An Observational Study. *International Journal of Public Health*, *67*(1), 1604978. https://doi.org/10.3389/IJPH.2022.1604978/BIBTEX

Perez, L. G., Dong, L., Beckman, R., & Meadows, S. O. (2021). Movement behaviors associated with mental health among US military service members. *Military Psychology*. https://doi.org/10.1080/08995605.2021.1987084

Rorem, D., Ezeugwu, V. E., Joly, V. J., Rasmussen, C., Carson, V., Tamana, S. K., Chikuma, J., Simons, E., Turvey, S., Mandhane, P. J., & Pei, J. (2024). Finding the balance: The influence of movement behaviours on childhood behaviour problems. *Mental Health and Physical Activity*, *26*, 100593. https://doi.org/10.1016/J.MHPA.2024.100593

Sampasa-Kanyinga, H., Chaput, J.-P., Goldfield, G. S., Janssen, I., Wang, J., Hamilton, H. A., Ferro, M. A., & Colman, I. (2021). The Canadian 24-hour movement guidelines and psychological distress among adolescents. *The Canadian Journal of Psychiatry*, *66*(7), 624–633. https://doi.org/10.1177/0706743720970863

Sampasa-Kanyinga, H., Colman, I., Dumuid, D., Janssen, I., Goldfield, G. S., Wang, J. L., Patte, K. A., Leatherdale, S. T., & Chaput, J. P. (2021). Longitudinal association between movement behaviours and depressive symptoms among adolescents using compositional data analysis. *PLoS ONE*, *16*(9), e0256867. https://doi.org/10.1371/JOURNAL.PONE.0256867

Sampasa-Kanyinga, H., Colman, I., Goldfield, G. S., Janssen, I., Wang, J., Tremblay, M. S., Barnes, J. D., Walsh, J. J., & Chaput, J.-P. (2021). 24-Hour Movement Behaviors and Internalizing and Externalizing Behaviors Among Youth. *Journal of Adolescent Health*, *68*, 969–977. https://doi.org/10.1016/j.jadohealth.2020.09.003

Sampasa-Kanyinga, H., Lien, A., Hamilton, H. A., & Chaput, J.-P. (2022a). Canadian 24-h Movement Guidelines, Life Stress, and Self-Esteem Among Adolescents. *Frontiers in Public Health*, *10*, 702162. https://doi.org/10.3389/fpubh.2022.702162

Sampasa-Kanyinga, H., Lien, A., Hamilton, H. A., & Chaput, J.-P. (2022b). The Canadian 24-hour movement guidelines and self-rated physical and mental health among adolescents. *Canadian Journal of Public Health*, *113*, 312–321. https://doi.org/10.17269/s41997-021-00568-7

Su, J., Wei, E., Clark, C., Liang, K., & Sun, X. (2022). Physical exercise, Sedentary Behaviour, Sleep and Depression Symptoms in Chinese Young Adults During the COVID-19 Pandemic: A Compositional Isotemporal Analysis. *International Journal of Mental Health Promotion*, *24*(5), 759–769. https://doi.org/10.32604/ijmhp.2022.020152

Sun, J., Jiang, X., & Wei, S. (2023). Combinations of physical activity, screen time and sleep, and their association with subjective wellbeing in children. *Complementary Therapies in Clinical Practice*, *51*, 101720. https://doi.org/10.1016/j.ctcp.2023.101720

Sun, Y., Luo, D., Guan, K., & Luo, X. (2024). Meeting 24-h movement behavior guidelines is associated with academic engagement, social-emotional functioning in obese/overweight youth. *Complementary Therapies in Clinical Practice*, *56*, 101863. https://doi.org/10.1016/j.ctcp.2024.101863

Tabaczynski, A., Courneya, K. S., & Trinh, L. (2020). Replacing sedentary time with physical activity and sleep: associations with quality of life in kidney cancer survivors. *Cancer Causes and Control*, *31*(7), 669–681. https://doi.org/10.1007/S10552-020-01308-X/TABLES/5

Tan, S. Y. X., Padmapriya, N., Bernard, J. Y., Toh, J. Y., Wee, H. L., Tan, K. H., Yap, F. K. P., Lee, Y. S., Chong, Y. S., Godfrey, K., Eriksson, J. G., Shek, L. P. C., Tan, C. S., Chong, M. F. F., & Müller-Riemenschneider, F. (2023). Cross-sectional and prospective associations between children’s 24-h time use and their health-related quality of life: a compositional isotemporal substitution approach. *The Lancet Regional Health - Western Pacific*, *41*. https://doi.org/10.1016/J.LANWPC.2023.100918/ATTACHMENT/5AD968BB-3128-4E4C-A9B2-4F8C48F0A453/MMC1.DOCX

Taylor, R. W., Haszard, J. J., Healey, D., Meredith-Jones, K. A., Taylor, B. J., & Galland, B. C. (2021). Adherence to 24-h movement behavior guidelines and psychosocial functioning in young children: a longitudinal analysis. *International Journal of Behavioral Nutrition and Physical Activity*, *18*(1), 110. https://doi.org/10.1186/S12966-021-01185-W/TABLES/5

Wang, S., Liang, W., Song, H., Su, N., Zhou, L., Duan, Y., Rhodes, R. E., Liu, H., Yang, Y. De, Lau, W. C. P., & Baker, J. S. (2023). Prospective association between 24-hour movement behaviors and mental health among overweight/obese college students: a compositional data analysis approach. *Frontiers in Public Health*, *11*, 1203840. https://doi.org/10.3389/FPUBH.2023.1203840/BIBTEX

Weatherson, K. A., Joopally, H., Wunderlich, K., Kwan, M. Y. W., Tomasone, J. R., & Faulkner, G. (2021). Post-secondary students’ adherence to the canadian 24-hour movement guidelines for adults: Results from the first deployment of the Canadian campus wellbeing survey (ccws). *Health Promotion and Chronic Disease Prevention in Canada*, *41*(6), 173–181. https://doi.org/10.24095/HPCDP.41.6.01

Yin, L., Li, F., Liu, P., Yin, Z., Yang, Z., Pi, L., & Gao, Z. (2024). Examining the relationship between meeting 24-hour movement behaviour guidelines and mental health in Chinese preschool children. *Frontiers in Pediatrics*, *12*, 1337158. https://doi.org/10.3389/FPED.2024.1337158/BIBTEX

Yuan, W., Zhang, Y., Yang, T., Cai, S., Chen, ; Ziyue, Song, ; Xinli, Dong, Y., Ma, J., Zhang, ; Xiuhong, & Song, Y. (2023). Association Between Combinations of 24-Hour Movement Behaviors and Depression Among Adolescents-Inner Mongolia Autonomous Region. *China CDC Weekly*, *40*(5). https://weekly.chinacdc.cn/

Zhang, R., Zhang, C.-Q., & Gu, D. (2023). Prospective associations between adherence to 24-hour movement guidelines and mental well-being in Chinese adolescents. *Journal of Sports Sciences*, *41*(19), 1735–1743. https://doi.org/10.1080/02640414.2023.2292422

Zhang, Y., Chi, X., Huang, L., Yang, X., & Chen, S. (2024). Cross-sectional association between 24-hour movement guidelines and depressive symptoms in Chinese university students. *PeerJ*, *12*(4), e17217. https://doi.org/10.7717/PEERJ.17217/SUPP-2

Zhang, Y., Pan, Y., Ma, Z., Wang, D., Zou, R., & Fan, F. (2023). Cross-sectional and longitudinal associations of adherence to the 24-hour movement guidelines with mental health problems among Chinese adolescents. *Journal of Psychosomatic Research*, *170*, 111352. https://doi.org/10.1016/j.jpsychores.2023.111352

Zhou, X., Shafrin Ahmad, N., Zamri Khairani, A., & Chen, Y. (2024). Association between meeting the 24-hour movement guideline and anxiety status in Chinese school-aged adolescents. *Environment and Social Psychology*, *9*(6). https://doi.org/10.54517/esp.v9i6.2509

Zhu, J. H., Shen, Z. Z., Liu, B. P., & Jia, C. X. (2024). Replacement of sedentary behavior with various physical activities and the risk of incident depression: a prospective analysis of accelerator-measured and self-reported UK Biobank data. *Social Psychiatry and Psychiatric Epidemiology*, 1–12. https://doi.org/10.1007/S00127-024-02708-Z/TABLES/4

Zhu, N., Guo, H., Ma, D., Wang, Q., Ma, J., & Kim, H. (2023). The Association between 24 h Movement Guidelines and Internalising and  Externalising Behaviour Problems among Chinese Preschool Children. *Children (Basel, Switzerland)*, *10*(7). https://doi.org/10.3390/children10071146

Zhu, X., Haegele, J. A., & Healy, S. (2019). Movement and mental health: Behavioral correlates of anxiety and depression among children of 6–17 years old in the U.S. *Mental Health and Physical Activity*, *16*, 60–65. https://doi.org/10.1016/j.mhpa.2019.04.002
