# Supplementary material for: 24-hour movement behaviours and mental health in non-clinical populations: A systematic review
Source: PLoS One. 2025 Jun 9;20(6):e0325445. doi: 10.1371/journal.pone.0325445 (PMC12148175; doi:10.1371/journal.pone.0325445)
Supplement: Attachment — Submitted filename: Response to Reviewers.docx [file pone.0325445.s006.docx]

Journal requirements:

When submitting your revision, we need you to address these additional requirements.

1. Please ensure that your manuscript meets PLOS ONE's style requirements, including those for file naming. The PLOS ONE style templates can be found at

<https://journals.plos.org/plosone/s/file?id=wjVg/PLOSOne_formatting_sample_main_body.pdf> and

<https://journals.plos.org/plosone/s/file?id=ba62/PLOSOne_formatting_sample_title_authors_affiliations.pdf>

Formatting has been edited according to the provided links.

2. In the online submission form you indicate that your data is not available for proprietary reasons and have provided a contact point for accessing this data. Please note that your current contact point is a co-author on this manuscript. According to our Data Policy, the contact point must not be an author on the manuscript and must be an institutional contact, ideally not an individual. Please revise your data statement to a non-author institutional point of contact, such as a data access or ethics committee, and send this to us via return email. Please also include contact information for the third party organization, and please include the full citation of where the data can be found.

All data can now be found in the supplementary materials.

3. As required by our policy on Data Availability, please ensure your manuscript or supplementary information includes the following:

A numbered table of all studies identified in the literature search, including those that were excluded from the analyses.

For every excluded study, the table should list the reason(s) for exclusion.

If any of the included studies are unpublished, include a link (URL) to the primary source or detailed information about how the content can be accessed.

A table of all data extracted from the primary research sources for the systematic review and/or meta-analysis. The table must include the following information for each study:

Name of data extractors and date of data extraction

Confirmation that the study was eligible to be included in the review.

All data extracted from each study for the reported systematic review and/or meta-analysis that would be needed to replicate your analyses.

If data or supporting information were obtained from another source (e.g. correspondence with the author of the original research article), please provide the source of data and dates on which the data/information were obtained by your research group.

If applicable for your analysis, a table showing the completed risk of bias and quality/certainty assessments for each study or outcome.  Please ensure this is provided for each domain or parameter assessed. For example, if you used the Cochrane risk-of-bias tool for randomized trials, provide answers to each of the signalling questions for each study. If you used GRADE to assess certainty of evidence, provide judgements about each of the quality of evidence factor. This should be provided for each outcome.

An explanation of how missing data were handled.

This information can be included in the main text, supplementary information, or relevant data repository. Please note that providing these underlying data is a requirement for publication in this journal, and if these data are not provided your manuscript might be rejected.

This data is now available in the supplementary materials (supplementary file 4).

Additional Editor Comments:

Thanks for your patience with the lengthy review time! In addition to the comments from the Reviewers, please consider the following comments. In particular, in light of other reviews I located (e.g., Kracht et al., 2024) it will be important for the authors to highlight the unique contributions of their review.

Thank you for pointing this out. Our review is the first to focus on mental health, rather than multiple health outcomes, with adult populations included. We have included a sentence in the introduction with references to highlight that most reviews have focused on physical health (line 80):

“Previous reviews have assessed the association between 24-hour movement and general health outcomes (7,12–14).”

We have also edited the aims sections to clarify our contribution to the literature.

1. Overall, the manuscript would benefit from thorough copy editing since this is not done by the journal if the manuscript is accepted for publication. I noted punctuation and grammar errors throughout. Specifically, in the results, findings from cited studies should be in the past tense (e.g., “Eleven of 12 studies that assessed meeting the guidelines…”).

Thank you, a final proof read has been conducted.

2. When referring to results of observational studies, please remove use of “effect” throughout the manuscript since only associations can be determined.

Thank you, we have edited accordingly throughout the manuscript.

3. The abstract lacks detail. The word limit is 300 for PLOS One so I suggest including additional information such as more detail on the search strategy. In addition, last part of the sentence on lines 30-31 seems like a fragment. Also, the authors mention that this research area is in its infancy but yet there are so many studies – that seems a bit contradictory.

This is true! Indeed it was in its infancy when we started but the updated search revealed a lot of new studies. We have rephrased to reflect the new status and added more details:

“There is increasing evidence that daily movement behaviours are associated with mental health. However the research into the relationship between 24-hour-movement and mental health, particularly in adults, is still to be systematically reviewed.”

4. Given that this is a narrative review, conclusions regarding age group differences may need to be tempered. While I appreciate the authors’ effort to review the evidence across all ages, perhaps a more focused approach on just one age group is warranted. Related to this, conclusions about the heterogeneity across studies is not surprising given the broad scope of this review. The authors cite the SR by Janssen and colleagues when describing the search strategy. It’s interesting to note that Janssen and colleagues focused just on adults and on studies that use compositional data analysis – so a much more focused approach.

The sentence on age group differences has been re-worded in the abstract.
We agree that the heterogeneity is not surprising but we feel it is still important to raise the point to explain why no meta-analysis was conducted. We have removed it from the certainty of evidence sentence to avoid the connotation that this is a limitation.
It is common practice to use a previous systematic review to assess the success of a search strategy and our informatics specialist advised us to use one which focused on analysing the composition of movement behaviours as this was an important inclusion criterion for us. Naturally our search found a much broader range of studies, but the important point was that our search found all of the studies included in Janssen et al.

5. Intro, Line 78: Please provide a reference for the opening statement. Similarly, please provide references for the statement on line 85 (“…a number of researchers have now begun to investigate…”)

A reference has been added at the start, thank you. We have used the current results as evidence of the extent of research into movement + mental health since we are the first to summarise this evidence.

6. Intro, Lines 98-101: The wording of this sentence could be improved (i.e., Across 10 studies [were they all cross-sectional?] included in the review, better mental health among children and youth was observed among those who met all three recommendations as compared with those who did not meet recommendations. A dose-response gradient was also observed…). Also, please cite the systematic review by Wilhite et al. (<https://doi.org/10.1093/aje/kwac212>) as they also investigated the relationship between movement behaviours and mental health (among other outcomes). There is also a recent systematic review by Zhao et al. (<https://doi.org/10.3389/fpubh.2024.1351972>).

The wording has been edited as suggested. The suggested citations have also now been included in the introduction (lines 101-116).

7. Line 105: Regarding the statement that no reviews on this topic have included adults, there is a recent umbrella review by Kracht and colleagues (<https://doi.org/10.1186/s44167-024-00064-6>) that should be cited, and the review by Rollo and colleagues covered the entire lifespan, as did a 2018 review by Grgic et al. (<https://doi.org/10.1186/s12966-018-0691-3>).

While the above reviews are important, they all focus on physical health with some mental health included. We have reworded to emphasise our focus on mental health outcomes (lines 119-121).

8. Intro, Line 107: Please use the same order for the populations as in the rest of the paper.

Edited.

9. Methods – Eligibility criteria – the subheading numbers for each PICOS item should be at the next level heading (e.g., 2.2.1 for Population, 2.2.2 for Intervention/exposure etc.)

Edited.

10. Please provide the age range for each population.

Good point - Included on lines 140-142:

“The age range definitions can vary slightly from study to study (supplementary materials) however typically children were 12 or under, adolescents 13-18 and adults >18 years.”

11. Methods – study designs: I am unclear what the authors mean by “…accounting for different combinations of recommendations met by participants in the analyses” – perhaps an example would help here?

Thank you for pointing out this unclear statement. An example of latent class analysis has been included here.

12. The publication year information on Lines 168-69 can be removed since it is already in the results.

Removed.

13. Line 180: Regarding percentage of each “gender” – I assume that most studies reported biological sex and not the social construct of gender, so please update accordingly.

Indeed true thank you. Some but not all report sex rather than gender so we have added sex to the sentence.

14. For the longitudinal studies, it would help to know the duration of follow-up. On a related note, while intervention studies/RCTs will be helpful, it seems that more longitudinal studies are needed. This was also highlighted as an area of future research in the umbrella review by Kracht and colleagues.

A range has been added to the manuscript (lines 245-246) and the duration for each study has been added to the respective notes in supplementary file 4.

Indeed longitudinal studies are very important and we have now highlighted this in the discussion (lines 586-588).

15. The discussion repeats the results in some sections (e.g., lines 435-443).

This is true, however we always attempted to summarise rather than repeat and the results highlighted in the discussion are to lead into a further discussion of the interpretation. Therefore we have chosen to leave this as is.

[Note: HTML markup is below. Please do not edit.]

Reviewers' comments:

Reviewer's Responses to Questions

**Comments to the Author**

1. Is the manuscript technically sound, and do the data support the conclusions?

The manuscript must describe a technically sound piece of scientific research with data that supports the conclusions. Experiments must have been conducted rigorously, with appropriate controls, replication, and sample sizes. The conclusions must be drawn appropriately based on the data presented.

Reviewer #1: Yes

Reviewer #2: Yes

2. Has the statistical analysis been performed appropriately and rigorously?

Reviewer #1: N/A

Reviewer #2: Yes

3. Have the authors made all data underlying the findings in their manuscript fully available?

The [PLOS Data policy](http://track.editorialmanager.com/CL0/http:%2F%2Fwww.plosone.org%2Fstatic%2Fpolicies.action%23sharing/1/010f019569a1609e-27447988-b44c-4530-8453-4d4f5df12d68-000000/AtVpU-x96VmFkPSEXpONubpNRFnlDfHZbshXWw6ovNA=201) requires authors to make all data underlying the findings described in their manuscript fully available without restriction, with rare exception (please refer to the Data Availability Statement in the manuscript PDF file). The data should be provided as part of the manuscript or its supporting information, or deposited to a public repository. For example, in addition to summary statistics, the data points behind means, medians and variance measures should be available. If there are restrictions on publicly sharing data—e.g. participant privacy or use of data from a third party—those must be specified.

Reviewer #1: Yes

Reviewer #2: Yes

4. Is the manuscript presented in an intelligible fashion and written in standard English?

PLOS ONE does not copyedit accepted manuscripts, so the language in submitted articles must be clear, correct, and unambiguous. Any typographical or grammatical errors should be corrected at revision, so please note any specific errors here.

Reviewer #1: Yes

Reviewer #2: Yes

5. Review Comments to the Author

Please use the space provided to explain your answers to the questions above. You may also include additional comments for the author, including concerns about dual publication, research ethics, or publication ethics. (Please upload your review as an attachment if it exceeds 20,000 characters)

Reviewer #1: Thank you for the opportunity to review this submission. Some suggestions as follows:

Thank you for the helpful suggestions!

- I recommend updating the keywords as they appear too similar to the title. Keywords should facilitate additional insights to the paper and expand its visibility

The keywords were chosen with support of the information specialist to be similar to, but not the same as, the title. However indeed some were the same as the title so we’ve now edited them to be somewhat broader.

- pg 3 line 42: not just the amount but the type/quality/frequency impact someone's health

Indeed true, we have edited accordingly. We have also added a statement in the discussion regarding this point (lines 541-545):

“Furthermore, recent studies suggest that some types of activity may have stronger impacts on mood and wellbeing than others. For example leisure-time, but not work-related physical activity, were related to happiness and stress, suggesting not only quantity, but also quality may be important (138). Future studies could more closely assess which types of PA, sleep etc. within the movement composition are most beneficial.”

- pg 3, line 50: what are you trying to get across? Yes we have finite time during the day but are you indicating "finite time to complete all work/life/health expectations"? it's an incomplete thought

The thought is completed in the following sentence (Consequently…). The sentences are split for emphasis, a stylistic writing choice we hope the reviewer will accept us leaving.

- pg 5, line 90-94: references needed to support your claims

Thank you, references have been added (line 98).

- pg 6, line 128 is redundant (MVPA is already explained as an acronym on pg 3, line 55)

True, thank you! Full terms deleted.

- pg 7, line 133: this is not a sentence. please rewrite.

The sentence has been edited.

- pg 7, line 137: why are these phrases underlined?

Because it was very important to highlight this aspect of the inclusion criteria. We have now italicised them rather than underline.

- why was the search frame limited to 10 years?

It was not limited but rather this was the range of the included studies. We realise this does not belong here and has been included in the results instead.

- since you exported to excel, how were duplicates removed? was this an automatic process?

As stated on line 192-193, duplicates were removed in a reference manager, not excel. Data extraction took place in excel (line 198).

- page 12 "emotional problems" needs to be rephrased. this isn't accurate and is negatively framing symptoms affiliated with mental health. suggest using "emotional disruptions" or something akin

“Emotional problems” is a common term utilised in the literature and therefore for conformity with common terminology we have chosen to keep it in. However we agree it would benefit from a definition and have added one (lines 271-272).

- your subheadings are confusing. "emotional problems" shows up on page 12, line 244 and then again on page 14, line 277. what are the differences? can they not be consolidated?

The results are split according to age category, so the first is for children and then next is for adolescents. They are separate sub-sections and hopefully this will be clear in the final article formatting.

- page 26, line 546. you need to rephrase this sentence because there are plenty of research studies on movement and mental health in adults - yours is focused on 24-hour movement in particular. additionally, the sentence written as-is, it's a runon sentence

Agreed thank you. We have included ’24-hour’.

- pg 26, line 554: you say you wanted to capture as much as possible, but you've limited the search to the last 10 years. why?

It was not limited but rather this was the range of the included studies. See comment above, we hope the confusion has been removed with our edit.

Reviewer #2: Dear Authors,

I would like to thank you for the opportunity to review your manuscript. Your study addresses an important and timely topic, examining the relationship between movement behaviors and mental health across different age groups. The paper provides a comprehensive review of existing literature and presents valuable findings that contribute to the growing body of research in this field.
Below, I provide detailed comments and suggestions that I believe will help enhance the clarity, methodological rigor, and impact of your manuscript. I hope these recommendations will be useful in refining your work.

Thank you for the helpful suggestions!

Major Comments
The introduction should more precisely formulate the research problem so that the reader clearly understands the main hypothesis or research question. Currently, the rationale for the study is somewhat broad and could be more explicitly defined.

Thank you, we have expanded this section to make the aims more explicit (lines 119-126). We have also split the paragraphs differently to increase emphasis on the novelty of our review.

Some topics in the introduction change abruptly and without clear transitions. For example, after discussing physical health (lines 41–60), the text suddenly shifts to methodological analysis methods (lines 61–76) and then to mental health (lines 77–88).
The description of movement behavior analysis methods (CoDA, ISM, lines 61–76) is overly detailed.

It is true that the topics change somewhat abruptly. We have edited the connecting statements to improve flow. We must partially disagree with the reviewer that the descriptions are too detailed. Since many of the results from the included studies come from these analyses, we believe this description allows non-expert readers to follow these results and allows us to simply refer to the analytical methods without going into details in the results section. Therefore we have chosen to leave most in, but have removed a small section that does indeed seem to be unnecessary (line 68).

The introduction presents data from studies on children and adolescents (lines 97–105), but their connection to adult mental health is not clearly established.
Since the article aims to examine associations across all age groups, it would be beneficial to balance the analysis of studies from different age groups and explicitly highlight their interconnections.

Indeed the issue is that more research has been conducted on children and adolescents. In particular it is important to describe what previous systematic reviews have found and these have only been conducted with young age groups, hence one aim of the current study being to include adults. With the reformatting of the paragraphs to increase emphasis on the lack of reviews in adults and the edited aims (thanks for this earlier suggestion), we hope this point will come across.

Although the literature search was conducted in PubMed, Scopus, and Embase (lines 156–169), there is no explanation for why other important databases, such as Web of Science, were not included.

Research shows that searching ≥2 databases is required for adequate coverage of the literature:

Ewald, H., Klerings, I., Wagner, G., Heise, T. L., Stratil, J. M., Lhachimi, S. K., ... & Nussbaumer-Streit, B. (2022). Searching two or more databases decreased the risk of missing relevant studies: a metaresearch study. *Journal of clinical epidemiology*, *149*, 154-164.

We have added this citation to the text to justify our method (line 177).

It is mentioned that most studies were observational ("Due to the observational nature of most studies, the quality was rather low", line 218), but there is no detailed analysis of the main quality shortcomings (e.g., selection bias, differences in measurement methods, or lack of control variables).

Thank you, this is a great point. We have included a summary of the risk of bias findings (lines 239-242).

The results indicate that certain movement behaviors are associated with better mental health, but there is no discussion of the potential mechanisms that could explain these associations. It would be beneficial to expand the discussion on the potential mechanisms explaining the relationship between movement behaviors and mental health.

Indeed the mechanisms are extremely interesting to consider. We have added a short speculation to the discussion (lines 546-551). We have chosen not to go into too much detail since the article is already quite long, but agree it’s definitely worth mentioning:

“Future research is also necessary to explore the working mechanisms underlying how movement behaviours influence mental health. It would be interesting to explore which common or specific factors of movement behaviours lead to improved mental health. Potentially, the working mechanisms known in psychotherapy research such as resource activation or mastery/coping/problem solving (139,140) could be working mechanisms also in the context of movement behaviours.”

The discussion section focuses more on data from adolescents and adults, while the findings from the children’s group are discussed only briefly, despite the fact that their results were more contradictory.

It is indeed interesting that their results were less clear. We have dedicated lines 477-488 to a discussion of the potential reasons for the contradictory results in children (in fact longer than the discussion of adolescent results), including four potential reasons and therefore we hope the reviewer will find is acceptable if we don’t add more. However, we are of course open to more suggestions that may explain the results as ours are surely not exhaustive.

It is noted that the findings may not be generalizable to African populations, but there is a lack of a broader discussion on how cultural and social factors might influence the association between movement behaviors and mental health.

Very true! Thanks for this point. We’ve added a short discussion of culture (lines 590-596).

Although the studies vary significantly in methodology, a more in-depth discussion of how this heterogeneity may influence the findings is needed.

This is discussed in lines 559-573. Here we discuss mental health as a primary vs secondary outcome, single item mental health measures vs scales, self-report vs objective PA measures, statistical technique. We concluded these are unlikely to have impacted the results (lines 571-573). We are happy to incorporate more suggestions.

Recent studies (e.g., Skurvydas et al., 2024, in BMC Public Health and PLOS ONE) highlight that leisure-time physical activity has a stronger impact on mood and well-being compared to work-related physical activity.

Thanks for pointing out this study. We have included it along with a short discussion of type vs simple quantity of PA (lines 541-546).

Overall, this study presents valuable insights into the relationship between movement behaviors and mental health, making a meaningful contribution to the field. However, addressing the aforementioned methodological and interpretative issues will significantly improve the manuscript’s clarity, coherence, and scientific rigor.
I hope these suggestions will be helpful in enhancing your paper. I appreciate your work and look forward to seeing the revised version.

Thanks so much for the constructive advice. We have endeavoured to incorporate it, whilst still trying to avoid the manuscript becoming even longer!
