# Supplementary material for: 24-hour movement behaviours and mental health in non-clinical populations: A systematic review
Source: PLoS One. 2025 Jun 9;20(6):e0325445. doi: 10.1371/journal.pone.0325445 (PMC12148175; doi:10.1371/journal.pone.0325445)
Supplement: Attachment — Submitted filename: Response to reviewers_revision 2.docx [file pone.0325445.s007.docx]

Response to reviewers: second revision

Thank you for addressing my comments and those of the 2 reviewers. My only remaining comment is that although the authors modified use of "effects" when referring to their own findings (except on line 36 in the abstract - "effects" needs to be changed), the same needs to be done when citing results of previous observational studies. For example, for the statement on lines 49-51 - were the cited reviews able to confirm evidence of "reciprocal effects" or just associations? Please check use of effect throughout the manuscript.

Thanks to the editor for their careful consideration of the manuscript and the amazing effort to find reviewers!

We have edited the line in the abstract as suggested.

On lines 49-51 we indeed mean effects. Changing one movement behaviour has an effect (increasing or decreasing) on other movement behaviours.

Line 69: effect is meant (effect of replacing one activity with another).

Line 73: edited.

Line 82: effect is meant (changes in mental wellbeing).

Line 94: edited.

Line 280: edited.

Sometimes we used the word effect not to refer to a directional effect of movement on mental health but rather to refer to statistical effects (i.e. a statistically significant model result). We have clarified this in relevant places by adding ‘statistical’ before the word effect.

Lines 319, 384, 392, 393, 426: referring to a statistical effect.

Line 397: effect is meant (effect of replacing one activity with another).

Line 407: effect is meant (effect of replacing one activity with another).

Line 427: effect of replacing one activity with another.

Line 458 & 463: edited to clarify statistical effect is meant – the remainder of the sentence refers to associations.

Line 543: refers to a RCT and therefore effect is correct.

Line 553: edited.

Line 575, 600: effect is correct.

Please review your reference list to ensure that it is complete and correct. If you have cited papers that have been retracted, please include the rationale for doing so in the manuscript text, or remove these references and replace them with relevant current references. Any changes to the reference list should be mentioned in the rebuttal letter that accompanies your revised manuscript. If you need to cite a retracted article, indicate the article’s retracted status in the References list and also include a citation and full reference for the retraction notice.

The reference list has been checked. Changes are visible in the tracked changes document and described below:

Rollo S, Antsygina O, Tremblay MS. The whole day matters: Understanding 24-hour movement guideline adherence and relationships with health indicators across the lifespan. J Sport Health Sci. 2020 Dec;9(6):493–510. – extra space removed.

Zhao H, Wu N, Haapala EA, Gao Y. Association between meeting 24-h movement guidelines and health in children and adolescents aged 5-17 years: a systematic review and meta-analysis. Front Public Health. 2024;12:1351972. – extra space removed.

Chao L, Ma R, Jiang W. Movement behaviours and anxiety symptoms in Chinese college students: A compositional data analysis. Front Psychol [Internet]. 2022 Sep 15 [cited 2024 Aug 22];13:952728. – incorrect link removed.

Ewald H, Klerings I, Wagner G, Heise TL, Stratil JM, Lhachimi SK, et al. Searching two or more databases decreased the risk of missing relevant studies: a meta research study. J Clin Epidemiol. 2022 Sep 1;149:154–64. – space added.

Fairclough SJ, Clifford L, Brown D, Tyler R. Characteristics of 24-hour movement behaviours and their associations with mental health in children and adolescents. Journal of activity, sedentary and sleep behaviors. 2023;2(1):11. – extra space removed.

Zhu N, Guo H, Ma D, Wang Q, Ma J, Kim H. The Association between 24 h Movement Guidelines and Internalising and Externalising Behaviour Problems among Chinese Preschool Children. Children (Basel). 2023 Jun;10(7). – extra space removed.

Kasai A, Shikano A, Tanaka R, Yoshinaga M, Noi S. School-aged children’s movement behaviours and subjective health complaints in Japan: a cross-sectional study during COVID-19 pandemic-related school closures and after school reopenings. BMC Public Health. 2024 Apr;24(1):1204. – extra space removed.

Dumuid D, Olds T, Lange K, Edwards B, Lycett K, Burgner DP, et al. Goldilocks Days: optimising children’s time use for health and well-being. J Epidemiol Community Health (1978) [Internet]. 2021 Mar;76(3):301–8. – incorrect link removed.

Burns RD, Bai Y, Pfledderer CD, Brusseau TA, Byun W. Movement behaviors and perceived loneliness and sadness within Alaskan adolescents. Int J Environ Res Public Health. 2020 Sep 2;17(18):1–13. – Alaskan capitalised.

Luo L, Zeng X, Cao Y, Hu Y, Wen S, Tang K, et al. The Associations between Meeting 24-Hour Movement Guidelines (24-HMG) and Mental Health in Adolescents-Cross Sectional Evidence from China. Int J Environ Res Public Health. 2023 Feb;20(4). – extra space removed.

García-Hermoso A, Ezzatvar Y, Ramírez-Vélez R, López-Gil JF, Izquierdo M. Trajectories of 24-h movement guidelines from middle adolescence to adulthood on depression and suicidal ideation: a 22-year follow-up study. Int J Behav Nutr Phys Act. 2022 Oct;19(1):135. – extra space removed.

Liu Y, Lin H, Zhang H, Zhang X, Yin S. Correlation analysis between physical activity and depressive tendencies among occupational groups: an isotemporal substitution approach. BMC Public Health. 2023 Nov;23(1):2241. – extra space removed.

Meneguci J, Galvão LL, Tribess S, Meneguci CAG, Virtuoso Júnior JS. Isotemporal substitution analysis of time between sleep, sedentary behavior, and physical activity on depressive symptoms in older adults: a cross-sectional study. Sao Paulo Med J. 2024;142(4):e2023144. – extra space removed.

Weatherson KA, Joopally H, Wunderlich K, Kwan MYW, Tomasone JR, Faulkner G. Post-secondary students’ adherence to the Canadian 24-hour movement guidelines for adults: Results from the first deployment of the Canadian campus wellbeing survey (ccws). Health Promotion and Chronic Disease Prevention in Canada. 2021;41(6):173–81. – Canadian capitalised.

Luo L, Cao Y, Hu Y, Wen S, Tang K, Ding L, et al. The Associations between Meeting 24-Hour Movement Guidelines (24-HMG) and Self-Rated Physical and Mental Health in Older Adults-Cross Sectional Evidence from China. Int J Environ Res Public Health. 2022 Oct;19(20). – extra space removed.

Le F, Yap Y, Yan N, Tung C, Bei Bei ·, Wiley JF. The Associations Between Daily Activities and Affect: A Compositional Isotemporal Substitution Analysis. Int J Behav Med [Internet]. 2021 [cited 2022 Jul 28];(3). Available from: <https://doi.org/10.1007/s12529-021-10031-z>. – A after colon capitalised.

Feng J, Lau PWC, Shi L, Huang WY. Longitudinal Shifts in Movement Behaviors during the COVID-19 Pandemic: Relations to Posttraumatic Stress Disorder among University Students. Int J Environ Res Public Health. 2022 Oct;19(20). – extra space removed.

Patton GC, Sawyer SM, Santelli JS, Ross DA, Afifi R, Allen NB, et al. Our future: a Lancet commission on adolescent health and wellbeing. Lancet [Internet]. 2016 Jun 6 [cited 2023 May 10];387(10036):2423. – incorrect link removed.

Kessler RC, Angermeyer M, Anthony JC, De Graaf R, Demyttenaere K, Gasquet I, et al. Lifetime prevalence and age-of-onset distributions of mental disorders in the World Health Organization’s World Mental Health Survey Initiative. World Psychiatry [Internet]. 2007 Oct [cited 2022 Jan 13];6(3):168–76. – incorrect link removed.

Carskadon MA. Sleep in Adolescents: The Perfect Storm. Pediatr Clin North Am [Internet]. 2011 Jun [cited 2023 May 10];58(3):637. – incorrect link removed.

Hidde MC, Lyden K, Henry K, Leach HJ. Reallocating time to physical activity and sleep: associations with quality of life in cancer survivors. Support Care Cancer. 2022 Sep;30(9):7527–34. – extra space removed.
